# A mucosal barrier injury related six gene transcriptomic signature associated with ulcerative colitis using machine learning and public transcriptomic data

**DOI:** 10.1371/journal.pone.0361599

**Published:** 2026-11-02

**Authors:** Sen He, Pingliang Sun, Xiaoxin Duan

**Affiliations:** 1 Graduate School, Guangxi University of Chinese Medicine, Nanning, Guangxi, China; 2 Department of Anorectal Diseases, The First Affiliated Hospital of Guangxi University of Chinese Medicine, Nanning, Guangxi, China; Penn State Health Milton S Hershey Medical Center, UNITED STATES OF AMERICA

## Abstract

Ulcerative colitis (UC) is characterized by epithelial barrier disruption, mucosal immune activation, and sustained intestinal inflammation. We developed a biologically interpretable public-transcriptomic signature using Gene Expression Omnibus datasets GSE75214, GSE38713, and GSE92415. A literature-curated panel of 35 mucosal barrier injury-related genes was integrated with differential expression, LASSO regression, random forest prioritization, and ridge logistic regression. Six genes (*LCN2*, *DEFB1*, *S100A8*, *MUC1*, *VIL1*, and *TJP3*) were retained as a locked transcriptomic score. Because the original feature-selection steps used the complete development cohort, we added 50 repetitions of stratified five-fold outer cross-validation. All preprocessing and ridge tuning were restricted to each outer-training fold; the locked score achieved a mean out-of-fold AUC of 0.9660 (SD 0.0031). A stricter sensitivity analysis that repeated differential-expression filtering, LASSO, random-forest ranking, deterministic six-gene selection, and ridge fitting within each outer-training fold achieved a mean out-of-fold AUC of 0.9600 (SD 0.0094). The refitted locked model retained discrimination in GSE38713 (AUC 0.8974, 95% CI 0.8040–0.9909) and GSE92415 (AUC 0.9894, 95% CI 0.9744–1.0000) when one development-derived cutoff was applied unchanged. The six-gene score did not significantly outperform *VIL1* alone in the development cohort (DeLong P = 0.4026). The signature should therefore be interpreted as a molecular pattern associated with UC mucosal inflammation and barrier remodeling rather than as a UC-specific clinical diagnostic test.

## 1. Introduction

Ulcerative colitis (UC) is a chronic relapsing inflammatory disorder of the colon characterized by recurrent mucosal inflammation, epithelial injury, and impaired intestinal homeostasis. Disease activity is commonly assessed through clinical symptoms, endoscopy, and histopathology, but these approaches may be invasive or resource-intensive and do not directly measure the molecular state of the mucosa [1–3]. Public transcriptomic cohorts provide an opportunity to examine molecular patterns associated with UC mucosal inflammation and to generate candidate signatures for biological and clinical investigation.

Epithelial barrier dysfunction is a central feature of UC pathobiology. Loss of epithelial integrity, altered mucus barrier function, antimicrobial peptide dysregulation, and tight junction remodeling can facilitate microbial stimulation and perpetuate immune activation [4–10]. A transcriptomic signature focused on barrier injury may therefore be more biologically interpretable than a purely data-driven marker panel, particularly if its components can be linked to epithelial structure, host-microbial interaction, and immune remodeling.

Previous studies have applied machine-learning approaches to transcriptomic data to distinguish UC from healthy controls and identify candidate molecular signatures [11–13]. These studies were primarily data-driven or used broad immune or transcriptome-wide feature spaces. In contrast, the present study restricted initial candidate selection to an a priori 35-gene mucosal barrier injury-related panel and combined complementary feature-prioritization methods with leakage-aware internal validation and locked, fixed-cutoff evaluation in two independent public cohorts. Our contribution therefore lies in the barrier-focused analytical organization and validation framework rather than in claiming discovery of previously unknown UC genes.

## 2. Materials and methods

### 2.1. Data acquisition and preprocessing

Gene expression profiles and clinical annotations were downloaded from the Gene Expression Omnibus database [14]. GSE75214 was used as the development cohort and included 97 colonic UC samples (74 annotated as active and 23 as inactive) and 11 normal colonic control samples. Crohn's disease samples and ileum-derived controls were excluded. GSE38713 and GSE92415 were used as independent public validation cohorts. GSE38713 contained 30 UC biopsies across deposited activity and biopsy-site categories and 13 non-inflammatory controls. GSE92415 contained 162 UC biopsies across study visits and 21 healthy controls. These heterogeneous annotations mean that the analysis evaluates a UC-associated mucosal expression pattern against healthy or non-inflammatory controls; it does not establish discrimination from Crohn's disease, infectious colitis, inactive disease, or other inflammatory conditions.

Series-matrix expression data and the corresponding platform annotation files were obtained for each cohort. Probe identifiers were converted to gene symbols, and expression values from multiple probes mapping to the same gene symbol were averaged. Each dataset was processed independently and the cohorts were never merged; consequently, cross-cohort batch correction was not applied. Development-cohort means and standard deviations were used for model scaling and were transferred unchanged to the external cohorts. Sample groups and disease-activity categories were assigned from explicit GEO phenotype fields rather than unrestricted keyword matching. No new participants, specimens, or wet-lab experiments were included in this secondary analysis of de-identified public data.

Ethical approval was not required because this study used only publicly available, de-identified transcriptomic datasets from the Gene Expression Omnibus database and did not involve newly recruited human participants, newly collected human tissue, or new animal experiments.

### 2.2. Differential expression analysis

Differential expression between UC and control samples in GSE75214 was analyzed using limma [15]. Benjamini-Hochberg correction was applied. Genes with an adjusted P value < 0.05 and an absolute log2 fold change > 1 were classified as DEGs. For the barrier-focused candidate set, adjusted P < 0.05 defined differential expression, while |log2 fold change| > 1 was additionally reported as a large fold change. Volcano plots and heatmaps were used for visualization.

### 2.3. Identification of mucosal barrier injury-related DEGs

An a priori curated mucosal barrier injury-related gene set was constructed before model development. Briefly, candidate genes were identified by reviewing representative literature on intestinal epithelial barrier dysfunction in IBD/UC and by querying functional categories directly related to barrier integrity, including tight junctions, mucins and mucus barrier, epithelial repair and regeneration, antimicrobial peptides and host defense, epithelial structure and brush-border organization, epithelial adhesion, and epithelial redox responses [4–10,16–21]. Search terms included “intestinal epithelial barrier”, “mucosal barrier”, “tight junction”, “mucin”, “mucus barrier”, “antimicrobial peptide”, “defensin”, “epithelial repair”, “epithelial adhesion”, “ulcerative colitis”, and “inflammatory bowel disease”. Genes were retained when they were repeatedly reported as canonical components or regulators of these barrier-related processes. Detectability of the curated genes was subsequently checked in the GSE75214 expression matrix. The final curated gene set contained 35 barrier-related genes and was finalized before differential expression and machine learning analyses to reduce data-driven selection bias. Differential expression of mucosal barrier injury-related genes was assessed using an adjusted P value < 0.05, and genes with |log2FC| > 1 were considered to have large fold changes. The complete curated gene set and functional classifications are provided in S3 Table in S1 Data.

### 2.4. Functional enrichment analysis

Functional enrichment analysis was performed using the gprofiler2 package [22] against a custom background comprising the 21,147 genes tested in GSE75214. Gene Ontology biological process, molecular function, and cellular component terms, together with KEGG, Reactome, and WikiPathways terms, were queried. The 563 upregulated genes, 303 downregulated genes, and 24 barrier-related DEGs were analyzed separately. The g:Profiler g:SCS multiple-testing correction was applied, and terms with adjusted P values < 0.05 were considered significant. Complete results are provided in S5-S7 Tables in S1 Data.

### 2.5. Machine-learning prioritization of candidate barrier-related genes

The mucosal barrier injury-related differentially expressed genes were used as candidate features. The initial discovery analysis applied LASSO logistic regression using glmnet and random forest ranking using MeanDecreaseGini [23,24]. LASSO retained seven candidates, including *DEFA5*; *DEFA5* was not retained in the locked signature because it ranked below the six leading random-forest features. The resulting locked genes were *LCN2*, *DEFB1*, *S100A8*, *MUC1*, *VIL1*, and *TJP3*. Because this original prioritization used the complete development cohort, its apparent training performance was considered potentially optimistic. We therefore added a prespecified full-pipeline nested-cross-validation sensitivity analysis. Within each outer-training fold, differential-expression filtering was repeated among the 35 genes, LASSO coefficients and random-forest importance values were separately rescaled to 0–1, their mean was used as a deterministic combined score, and the six highest-scoring genes were passed to ridge regression. No outer-test sample contributed to filtering, scaling, feature ranking, tuning, or fitting.

### 2.6. Six-gene transcriptomic score construction and public-cohort validation

The six published genes were evaluated as a locked transcriptomic score. Fifty repetitions of stratified five-fold outer cross-validation were performed in GSE75214. Within every outer split, z-score parameters, class weights, the ridge penalty, and model coefficients were estimated using only the outer-training samples. The ridge penalty (alpha = 0) was selected by stratified five-fold inner cross-validation, and predictions were generated only for the held-out outer-test samples. AUC was calculated separately for every repeated outer partition and summarized by its mean, standard deviation, median, range, and empirical 2.5th-97.5th percentiles. In parallel, the complete feature-selection pipeline described in Section 2.5 was repeated within each outer-training fold as a sensitivity analysis. After internal evaluation, a final locked six-gene ridge model was refitted using the complete GSE75214 development cohort.

For external validation, the development-derived gene-wise means and standard deviations, final ridge coefficients, and one development-derived Youden cutoff were applied unchanged to GSE38713 and GSE92415. Discrimination was evaluated using ROC analysis with pROC [25], and AUCs with 95% CIs were calculated [26]. The fixed cutoff was used to calculate sensitivity, specificity, accuracy, PPV, and NPV [27]. The external datasets were processed independently and were not used for feature selection, model tuning, coefficient estimation, or cutoff selection.

Decision curve analysis was performed as an exploratory assessment of the potential usefulness of the transcriptomic score for public-cohort classification. Net benefit was calculated across threshold probabilities from 0.01 to 0.80 and compared with treat-all and treat-none strategies. These analyses were interpreted as computational evidence only and not as proof of clinical utility.

Calibration curves were generated to assess the agreement between predicted probabilities and observed UC status. Brier scores were calculated to quantify overall prediction error.

### 2.7. Immune marker score and correlation analysis

Immune cell marker gene sets were curated for neutrophils, macrophages, monocytes, CD8 + T cells, CD4 + T cells, regulatory T cells, Th17 cells, B cells, plasma cells, natural killer cells, and dendritic cells. For each sample, marker genes were standardized gene-wise and averaged to obtain an immune marker score. Prespecified marker sets are provided in S11 Table in S1 Data. UC-control differences were assessed using two-sided Wilcoxon rank-sum tests with Benjamini-Hochberg correction across the 11 scores; complete results are provided in S12 Table in S1 Data.

Spearman correlation analysis was performed to assess associations between signature-gene expression and immune marker scores. Correlation coefficients and Benjamini-Hochberg-adjusted P values for all 66 gene-score comparisons are provided in S13 Table in S1 Data.

### 2.8. Statistical analysis

Continuous variables between two groups were compared using the Wilcoxon rank-sum test. Multiple-testing correction used the Benjamini-Hochberg method [28]. Correlations were evaluated using Spearman coefficients. Correlated AUCs for the locked six-gene score and *VIL1* alone were compared using the paired DeLong test [26]. Two-sided P values < 0.05 were considered statistically significant. Empirical percentiles across repeated cross-validation partitions describe partition-to-partition variability and were not interpreted as conventional confidence intervals.

### 2.9. Reporting guideline

Applicable items from the TRIPOD+AI statement were followed for transparent reporting of model development, validation, and interpretation [29]. A completed TRIPOD+AI checklist is provided as an additional file.

## 3. Results

### 3.1. Public-cohort study design and data processing

The overall workflow is shown in Fig 1. GSE75214 was used as the development cohort and contained 97 colonic UC samples (74 active and 23 inactive) and 11 normal colonic controls. GSE38713 (30 UC and 13 controls) and GSE92415 (162 UC and 21 controls) were retained as independent public validation cohorts. Probe-to-gene conversion and phenotype assignment were performed independently within each dataset before downstream analysis. Dataset characteristics are summarized in Table 1.

**Table 1:** Dataset information included in this study.

Dataset	Role	Control samples	UC samples	Main use
GSE75214	Development cohort	11	97 (74 active; 23 inactive)	Discovery analysis and repeated nested cross-validation
GSE38713	External validation cohort	13	30	Independent fixed-cutoff validation
GSE92415	External validation cohort	21	162	Independent fixed-cutoff validation

Note: GSE75214 included 97 colonic UC samples (74 active and 23 inactive) and 11 normal colonic controls. Crohn's disease samples and ileum-derived controls were excluded.

**Fig 1:**
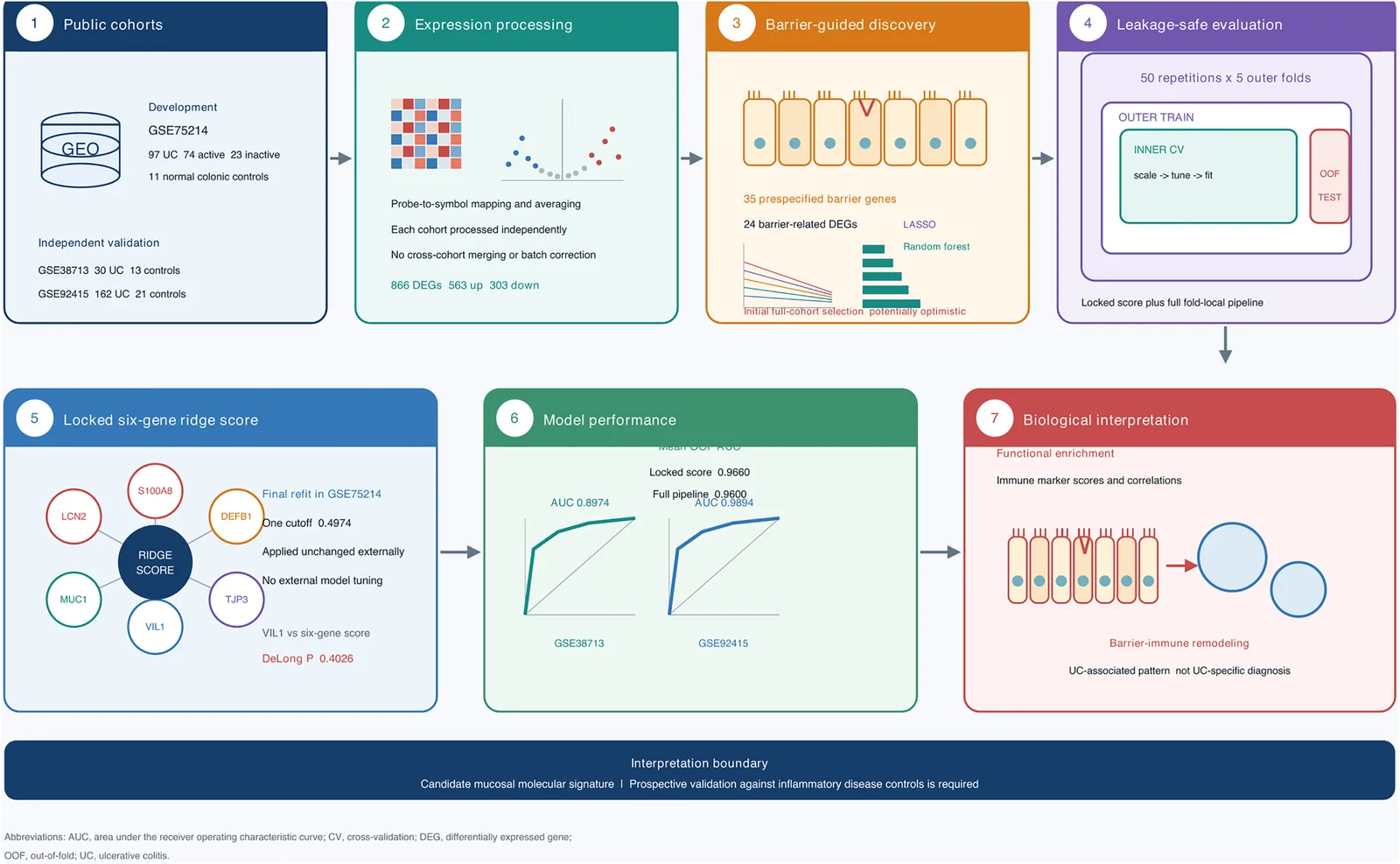
Study workflow. The workflow includes cohort-specific preprocessing, differential-expression analysis, prespecified barrier-gene filtering, initial machine-learning prioritization, repeated nested cross-validation, locked-score evaluation, fold-local feature-selection sensitivity analysis, fixed-cutoff external validation, and immune marker analysis.

### 3.2. UC mucosa exhibits immune activation and barrier dysfunction

Differential-expression analysis identified 866 DEGs between UC and control samples in GSE75214, including 563 upregulated and 303 downregulated genes. The volcano plot shows the overall distribution of these genes, and the heatmap of leading DEGs demonstrates distinct transcriptomic profiles between UC and control samples (Fig 2A,2B).

**Fig 2:**
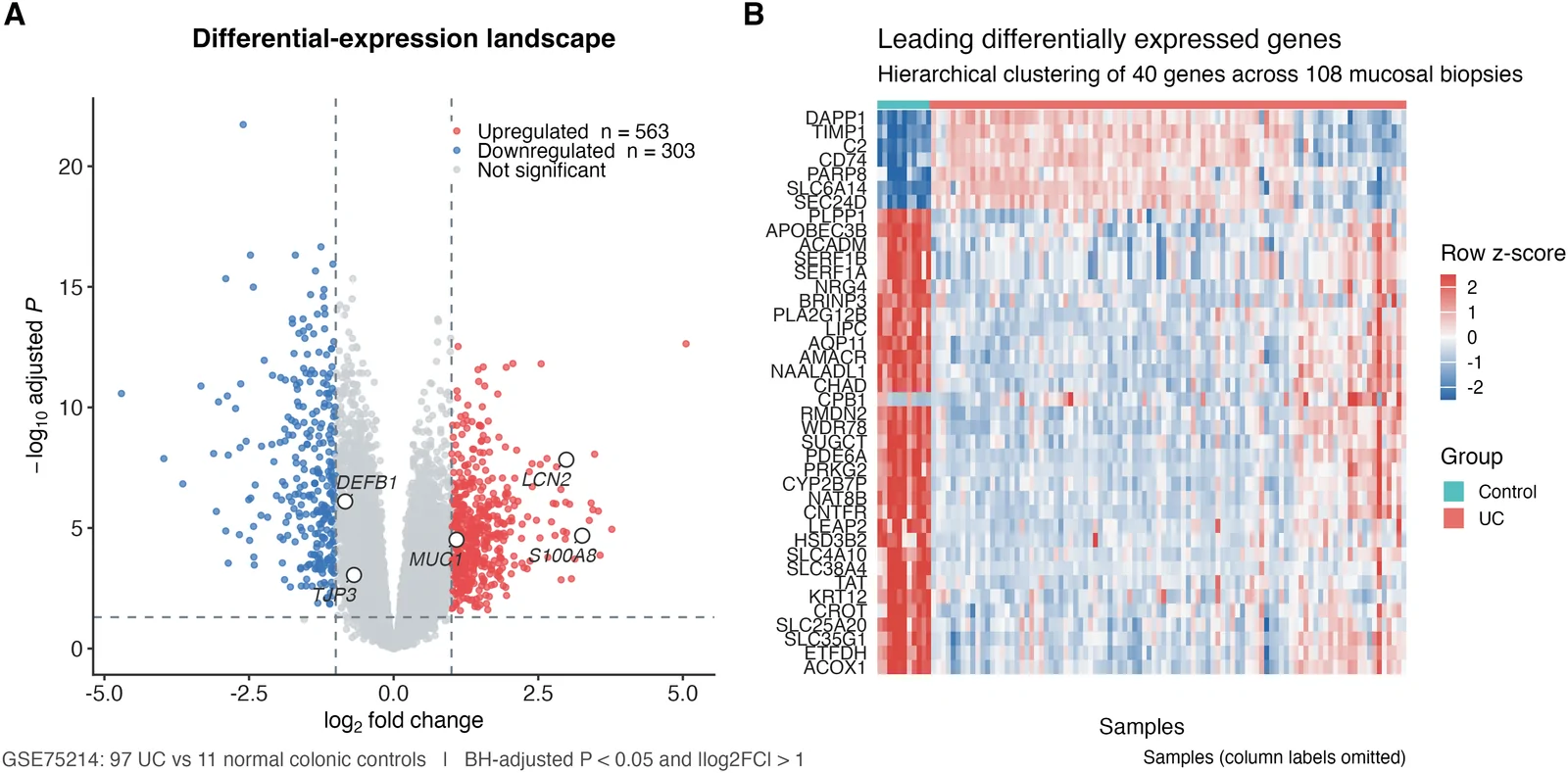
Differential-expression landscape between UC and control mucosal biopsies in GSE75214. (A) Volcano plot showing genes meeting the adjusted P-value and fold-change criteria. (B) Heatmap of leading differentially expressed genes. GSE75214 contained 97 colonic UC samples, including active and inactive disease, and 11 normal colonic controls.

### 3.3. Functional enrichment analysis of DEGs

Functional enrichment was performed separately for upregulated and downregulated genes. Upregulated genes were predominantly associated with immune and inflammatory processes, including immune response, defense response, and response to external stimuli. Downregulated genes were predominantly associated with metabolic and transport processes, including lipid and small-molecule metabolism and transmembrane transport. These patterns indicate concurrent immune-inflammatory activation and impaired epithelial metabolic and transport functions in UC mucosa (S1A,B Fig).

### 3.4. Barrier injury related genes are dysregulated in UC mucosa

All 35 curated mucosal barrier injury-related genes were detectable in GSE75214, and 24 differed significantly between UC and controls. Seventeen were upregulated and seven were downregulated; 16 of the 24 also showed |log2 fold change| > 1. The heatmap and representative boxplots illustrate coordinated but heterogeneous remodeling of inflammatory, antimicrobial, and epithelial structural components (Fig 3A,3B; S3 and S4 Tables in S1 Data).

**Fig 3:**
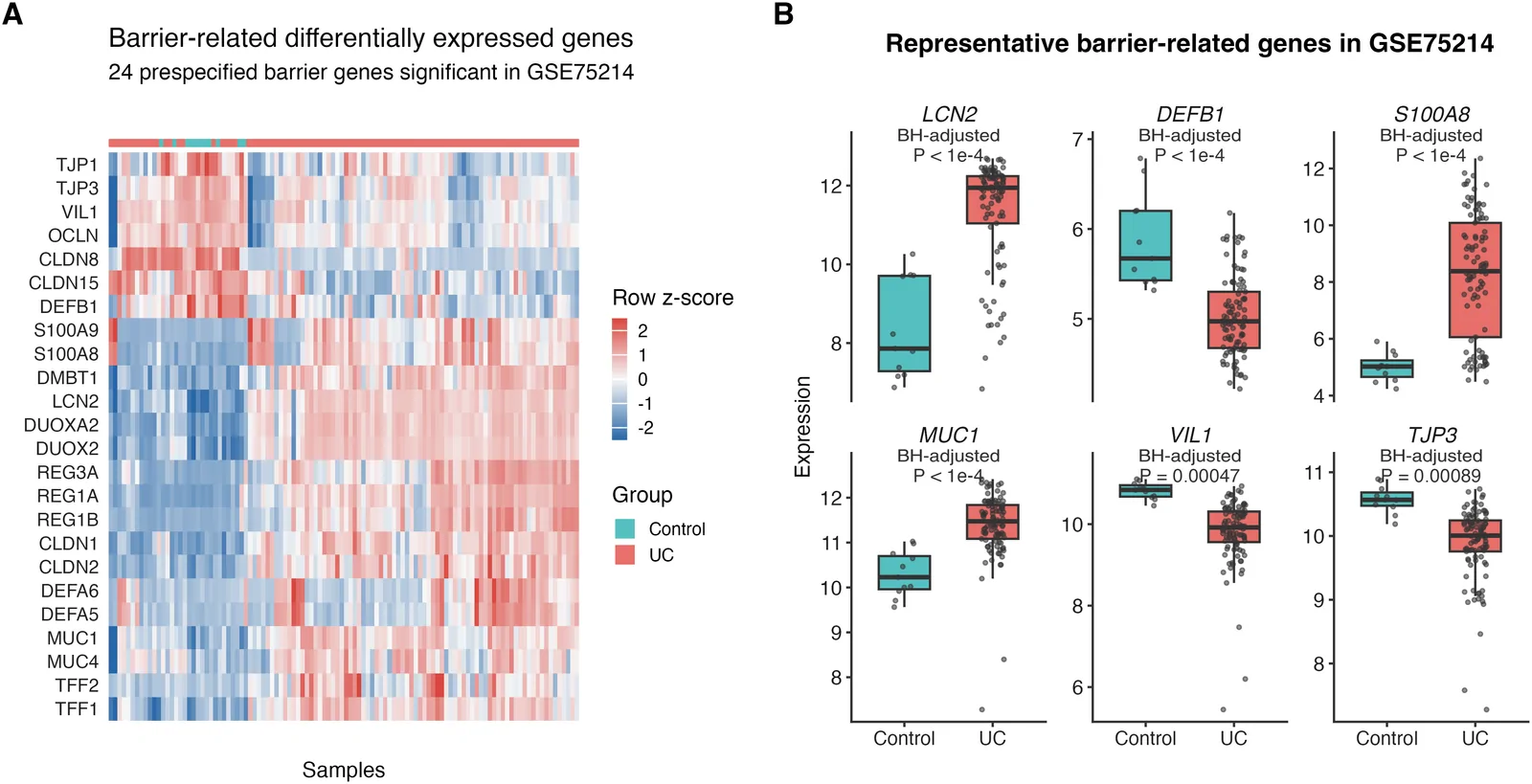
Expression characteristics of mucosal barrier injury-related genes in UC. (A) Heatmap showing barrier-related gene expression in GSE75214, with samples annotated as Control or UC. (B) Boxplots comparing representative genes between groups. The panels describe association with UC mucosal status and should not be interpreted as evidence of UC specificity.

Functional enrichment of the barrier-related differentially expressed genes highlighted antimicrobial humoral responses, antimicrobial peptide-mediated defense, responses to biotic stimuli, and host-organism interactions. These findings connect the prespecified barrier gene space to mucosal antimicrobial defense and epithelial-immune interactions without establishing causal relationships (S2 Fig).

### 3.5. Machine learning prioritized six barrier-related candidate genes

In the initial complete-cohort discovery analysis, LASSO retained *LCN2*, *DEFB1*, *S100A8*, *MUC1*, *VIL1*, *TJP3*, and *DEFA5*, while random forest placed the first six genes above *DEFA5*; *DEFA5* was therefore treated as a LASSO-only candidate and excluded from the locked score (Fig 4A,4B). In the leakage-safe sensitivity analysis, feature selection was repeated within each outer-training fold using the deterministic combined LASSO/random-forest score. The most frequently selected genes were *OCLN* (99.6%), *VIL1* (96.8%), *TJP1* (94.8%), *MUC1* (86.4%), *S100A8* (53.2%), and *DEFB1* (46.0%). The exact published six-gene composition was not uniformly recovered, indicating that feature identity was less stable than discrimination performance.

**Fig 4:**
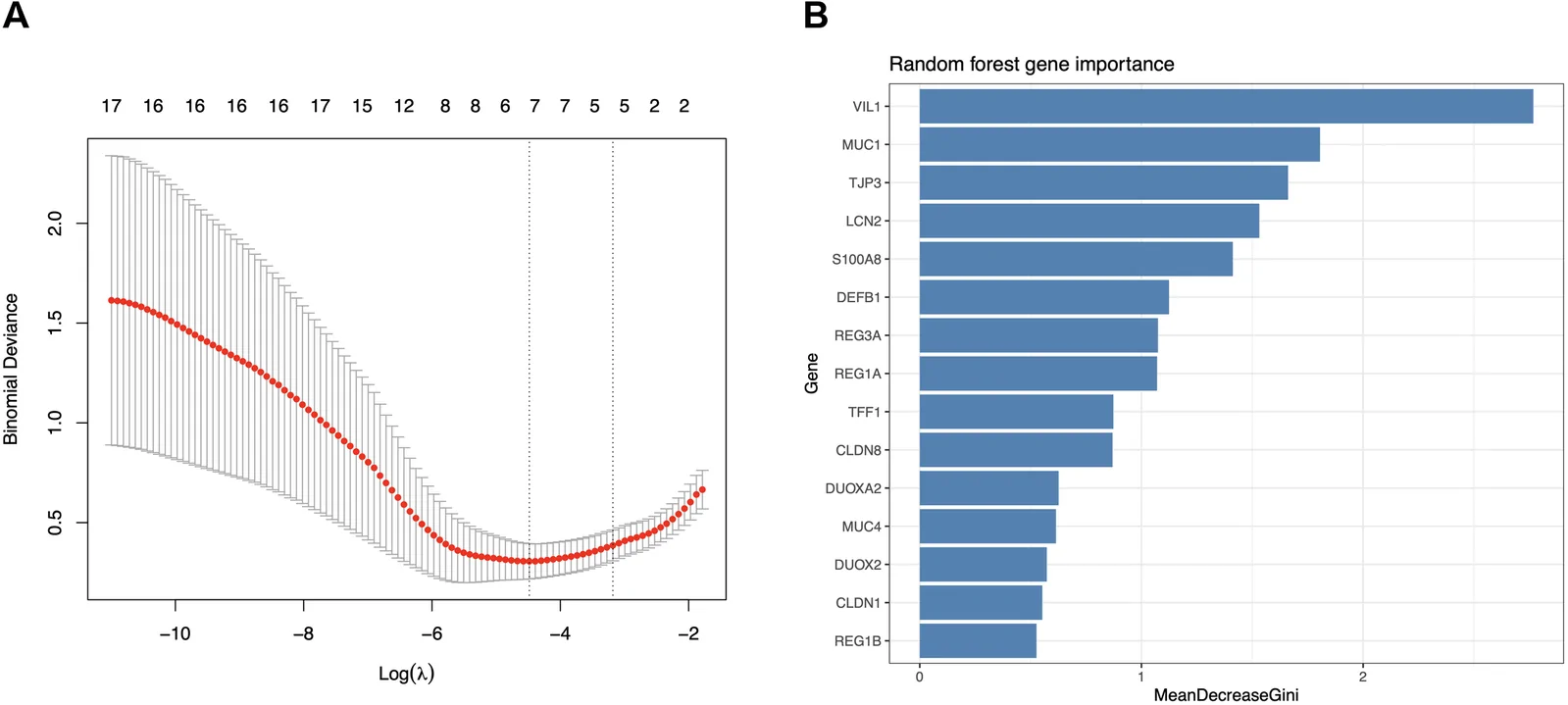
Initial machine-learning prioritization and leakage-safe sensitivity analysis. A) LASSO selection in the complete development cohort. (B) Random-forest MeanDecreaseGini ranking. These complete-cohort panels describe the original discovery analysis. In the added nested analysis, differential-expression filtering, LASSO, random forest, deterministic six-gene ranking, and ridge fitting were repeated separately within every outer-training fold.

### 3.6. Discrimination and calibration of the six-gene transcriptomic score

In the complete GSE75214 cohort, individual-gene AUCs were 0.9288 for *LCN2*, 0.9007 for *DEFB1*, 0.9138 for *S100A8*, 0.9316 for *MUC1*, 0.9616 for *VIL1*, and 0.9213 for *TJP3* (Table 2). *VIL1* had the highest single-gene AUC. The apparent AUC of the fitted six-gene score was 0.9747, but its improvement over *VIL1* alone was not statistically significant (paired DeLong test, P = 0.4026). Accordingly, the multigene score is interpreted as a broader representation of barrier-immune biology rather than a demonstrated statistical improvement over *VIL1* alone.

**Table 2:** Discrimination performance of individual candidate genes in the GSE75214 development cohort.

Gene	AUC	95% CI	Sensitivity	Specificity	Cutoff
*LCN2*	0.9288	0.8726-0.9850	0.8041	1.0000	10.2916
*DEFB1*	0.9007	0.8337-0.9676	0.7526	1.0000	5.3096
*S100A8*	0.9138	0.8485-0.9790	0.7629	1.0000	5.9235
*MUC1*	0.9316	0.8748-0.9884	0.7732	1.0000	11.0256
*VIL1*	0.9616	0.9245-0.9987	0.8454	1.0000	10.4468
*TJP3*	0.9213	0.8505-0.9921	0.9175	0.8182	10.4551

Abbreviations: AUC, area under the receiver operating characteristic curve; CI, confidence interval. Sensitivity and specificity were calculated according to the optimal cutoff determined by the Youden index.

After correcting the validation procedure, the locked six-gene ridge score achieved a mean out-of-fold AUC of 0.9660 (SD 0.0031; empirical 2.5th-97.5th percentiles, 0.9597–0.9700) across 50 repetitions of stratified five-fold outer cross-validation. The stricter fold-local analysis that repeated differential-expression filtering, LASSO, random forest, six-gene selection, and ridge fitting achieved a mean out-of-fold AUC of 0.9600 (SD 0.0094; empirical percentiles, 0.9409–0.9756). In external validation, the refitted locked model achieved an AUC of 0.8974 (95% CI 0.8040–0.9909) in GSE38713 and 0.9894 (95% CI 0.9744–1.0000) in GSE92415. With the single GSE75214-derived cutoff of 0.4974, sensitivity and specificity were 0.7000 and 1.0000 in GSE38713 and 0.9136 and 0.9048 in GSE92415, respectively (Table 3; Fig 5A,5B). Ridge-penalty tuning and the repeated nested-cross-validation results are shown in Fig 6A,6B.

**Table 3:** Public-cohort validation performance of the ridge regression-based six-gene transcriptomic score using the training-derived cutoff.

Dataset	AUC	95% CI	Cutoff	Sens.	Spec.	Acc.	PPV	NPV	Ctrl	UC
GSE38713	0.8974	0.8040-0.9909	0.4974	0.7000	1.0000	0.7907	1.0000	0.5909	13	30
GSE92415	0.9894	0.9744-1.0000	0.4974	0.9136	0.9048	0.9126	0.9867	0.5758	21	162

Genes used in the locked ridge score: LCN2, DEFB1, S100A8, MUC1, VIL1, and TJP3. A single cutoff of 0.4974 was derived in GSE75214 and applied unchanged to both public validation cohorts. Abbreviations: AUC, area under the receiver operating characteristic curve; CI, confidence interval; PPV, positive predictive value; NPV, negative predictive value; Ctrl, control samples.

**Fig 5:**
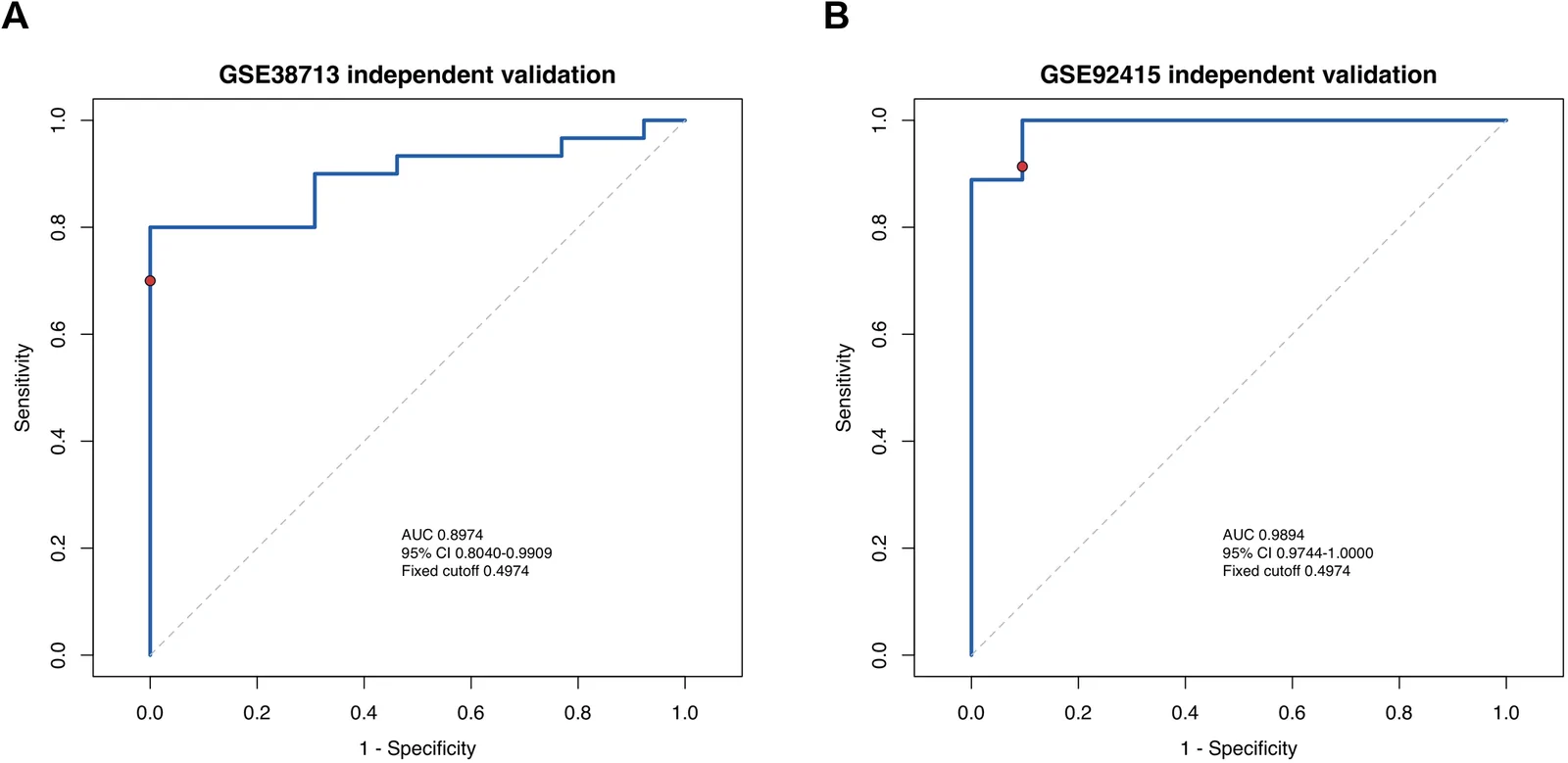
Independent public-cohort validation of the locked six-gene score. (A) ROC curve in GSE38713. (B) ROC curve in GSE92415. Both datasets were excluded from feature selection, tuning, coefficient estimation, and cutoff selection; the GSE75214-derived cutoff was applied unchanged.

**Fig 6:**
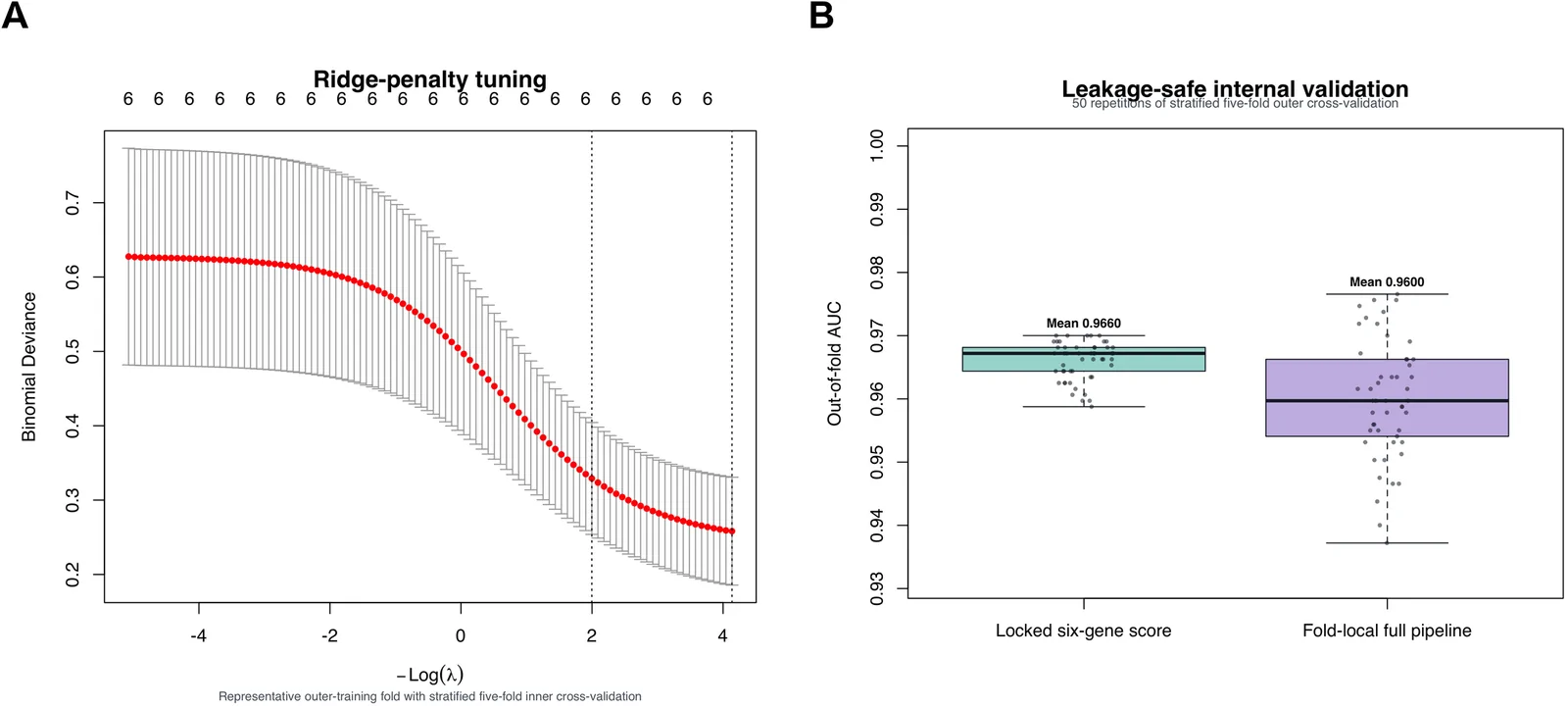
Ridge-model tuning and leakage-safe internal validation. (A) Inner five-fold cross-validation used to select the ridge penalty within each outer-training set. (B) Distribution of out-of-fold AUCs across 50 repetitions of stratified five-fold outer cross-validation for the locked six-gene score and the complete fold-local feature-selection pipeline.

Decision curve analysis was retained as an exploratory assessment of the six-gene score for UC-control classification in the public cohorts. The score showed positive net benefit across portions of the evaluated threshold range, but these retrospective computational results do not demonstrate clinical decision utility and should not be extrapolated to differential diagnosis (Fig 7).

**Fig 7:**
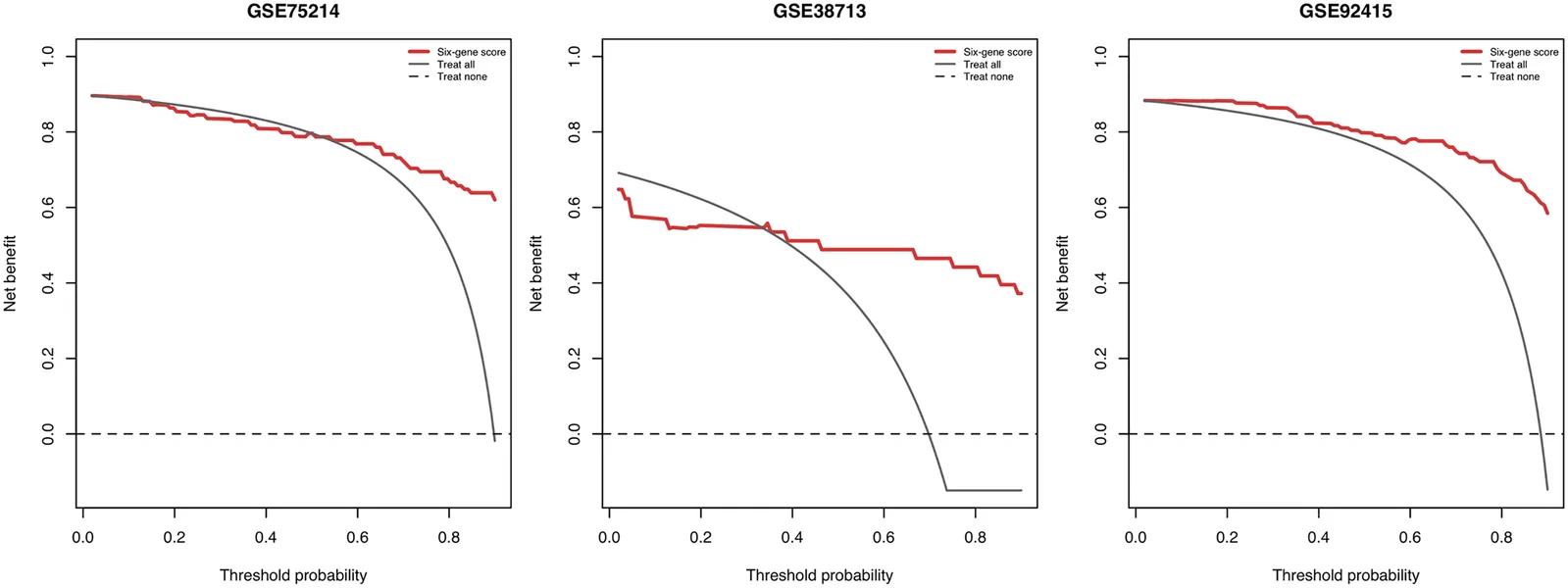
Decision curve analysis of the ridge regression-based six-gene transcriptomic score. (A) Decision curve analysis of the score in the GSE75214 training cohort. (B) Decision curve analysis of the score in the GSE38713 validation cohort. (C) Decision curve analysis of the score in the GSE92415 validation cohort. The score showed a positive net benefit compared with the treat-none strategy across a broad range of threshold probabilities and generally provided additional net benefit over the treat-all strategy, particularly at moderate to high threshold probabilities.

Calibration varied across cohorts, with less stable probability estimates in GSE38713 than in GSE92415. Because the cohorts differed in platform, disease activity, treatment exposure, biopsy status, and sampling schedule, probability calibration should not be assumed to transfer directly to new clinical settings. These analyses are exploratory and require prospective recalibration (S3 Fig).

### 3.7. Immune marker score analysis revealed immune activation in UC

Immune marker score analysis showed higher plasma-cell, neutrophil, monocyte, CD4 + T-cell, regulatory T-cell, CD8 + T-cell, B-cell, and macrophage marker scores in UC than in controls. Plasma-cell, neutrophil, and monocyte scores showed particularly strong differences after multiple-testing correction. Dendritic-cell, natural-killer-cell, and Th17 marker scores did not differ significantly in this marker-score analysis (Fig 8A,8B; S12 Table in S1 Data).

**Fig 8:**
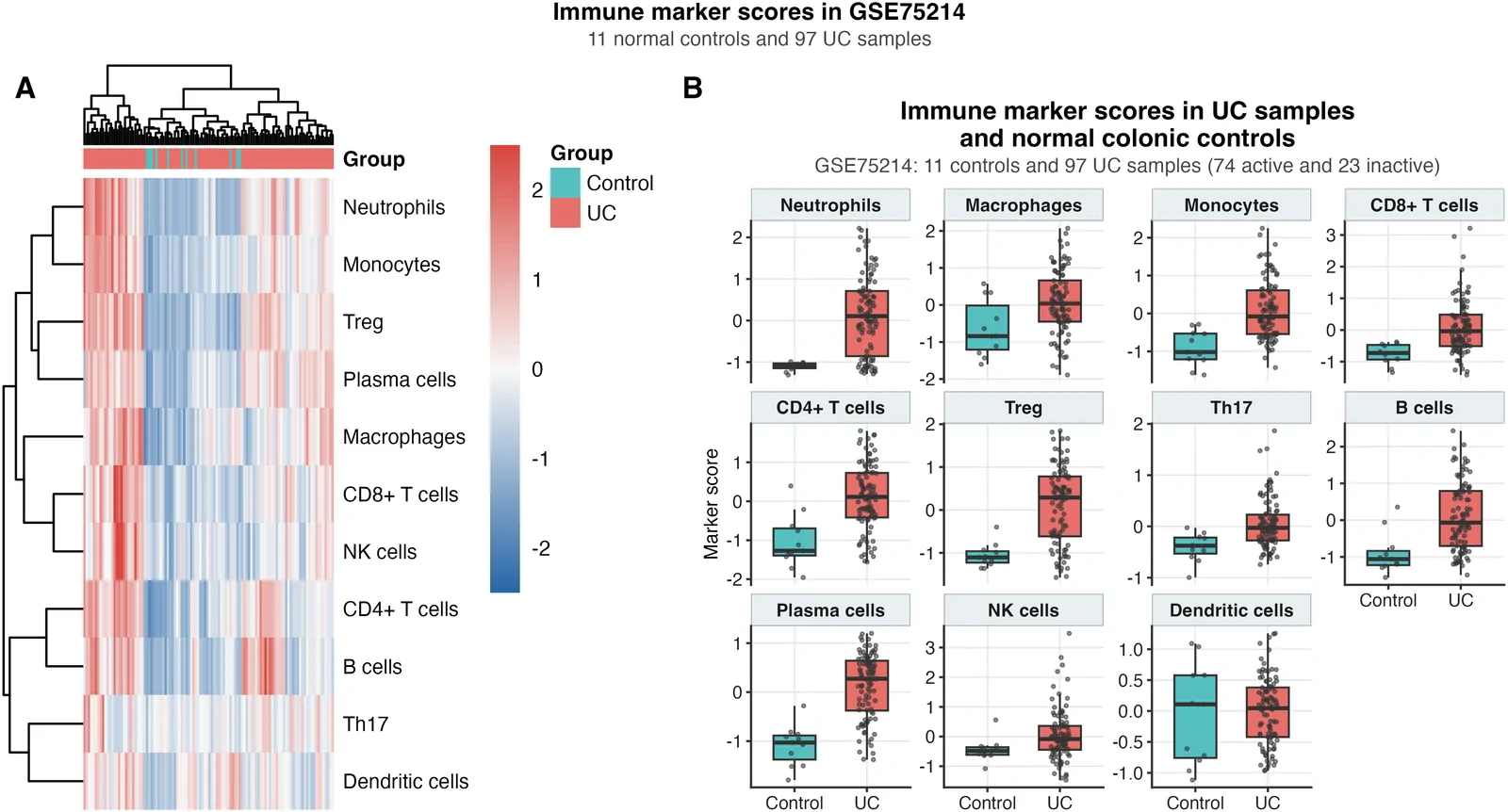
Immune marker score analysis in GSE75214 mucosal biopsies. A) Heatmap of standardized immune marker scores across all 108 samples (11 normal controls and 97 UC samples). (B) Boxplots comparing the same scores between 97 UC samples (74 active and 23 inactive) and 11 normal controls; the comparison was UC versus control. Scores are transcript-based surrogates and not direct cell measurements.

### 3.8. Signature genes were associated with immune marker scores

Spearman correlation analysis revealed distinct associations between signature genes and immune marker scores. *S100A8* and *LCN2* showed an inflammation-related pattern: *S100A8* correlated positively with monocyte, neutrophil, plasma-cell, regulatory T-cell, macrophage, CD4 + T-cell, and CD8 + T-cell scores, while *LCN2* correlated positively with plasma-cell, regulatory T-cell, neutrophil, and monocyte scores. In contrast, *VIL1* and *TJP3* were broadly negatively correlated with several immune scores; *MUC1* showed weaker, cell-type-dependent associations, and *DEFB1* correlated negatively with several scores (Fig 9; S13 Table in S1 Data). These associations support a barrier-immune imbalance pattern in UC mucosa but do not establish cellular origin or causality.

**Fig 9:**
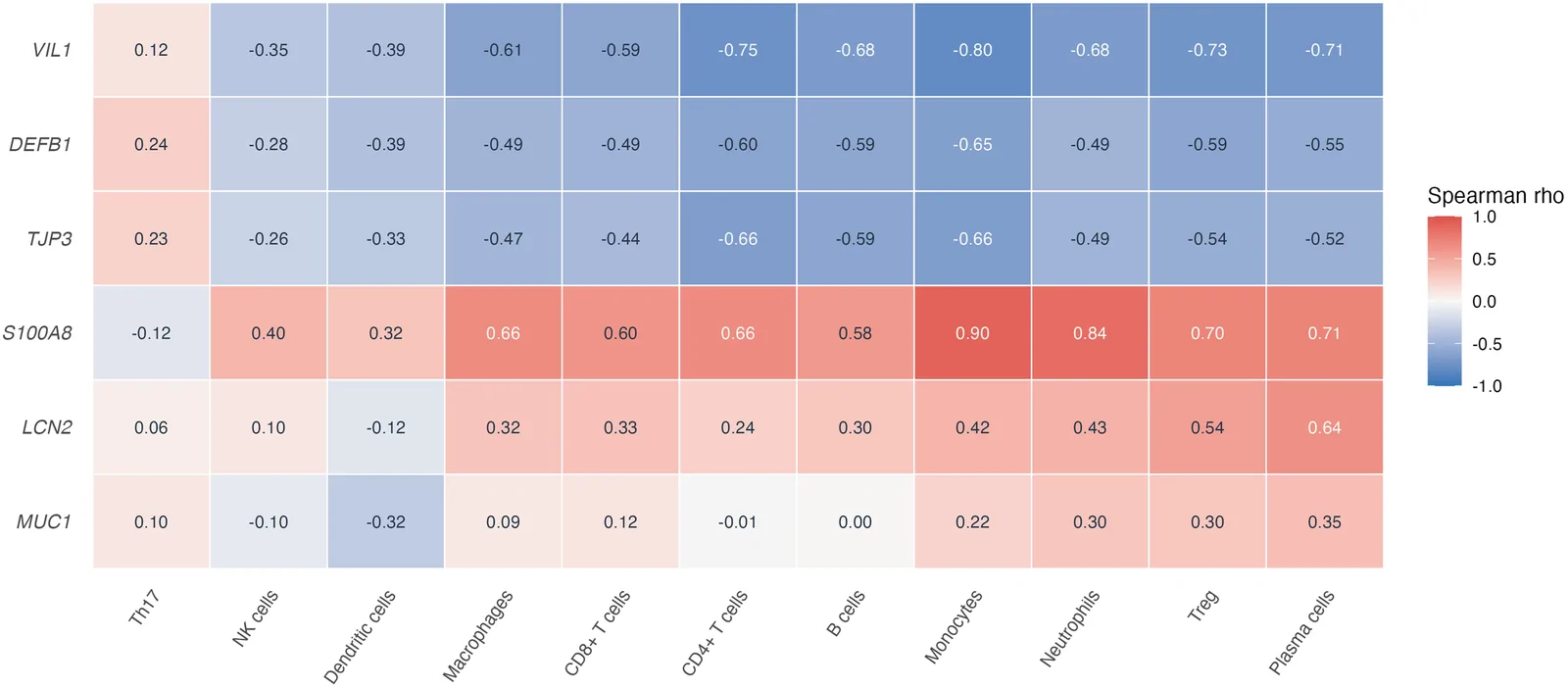
Signature-gene correlations with immune marker scores. The heatmap shows Spearman correlation coefficients between expression of the six signature genes and immune marker scores across all 108 GSE75214 samples. These transcript-derived associations do not identify cellular origin or causality.

## 4. Discussion

This study developed and evaluated a biologically interpretable six-gene public-transcriptomic signature associated with UC mucosal barrier injury and immune remodeling. The locked score consisted of *LCN2*, *DEFB1*, *S100A8*, *MUC1*, *VIL1*, and *TJP3*. Repeated nested cross-validation addressed optimistic internal evaluation in the original workflow, while two public cohorts provided independent validation. The score is not presented as a UC-specific clinical diagnostic test.

A key strength of this work is the use of an a priori mucosal barrier injury-related gene panel. UC involves epithelial barrier damage, mucus barrier alteration, host-microbial interaction, and sustained mucosal immune activation. The enrichment of barrier-related DEGs in antimicrobial humoral response and antimicrobial peptide-mediated immune response supports the biological relevance of the selected gene space [4–10,16–19]. This strategy may reduce the risk of selecting statistically strong but biologically opaque markers and helps make the final signature more accessible to biologists and clinicians.

The signature genes represent complementary inflammatory and epithelial processes. *LCN2* and *S100A8* are associated with innate immune activation [30,31] and correlated positively with several immune marker scores, particularly neutrophil and monocyte scores. These findings are consistent with mucosal inflammatory activity but cannot determine whether the measured expression originated from epithelial cells, infiltrating immune cells, or both.

*MUC1*, *VIL1*, and *TJP3* represent epithelial barrier structure, mucosal homeostasis, and junction-related biology [4,6,9,16,17,20]. Their altered expression may reflect barrier remodeling or compensatory epithelial responses. *DEFB1* is related to antimicrobial host defense and epithelial-microbial interactions [18,19]. Together, the opposing correlation patterns of inflammatory and epithelial structural genes support biological organization across multiple barrier-immune dimensions, even though the multigene AUC was not significantly greater than that of *VIL1* alone. This integrative biological interpretation is summarized in Fig 10.

**Fig 10:**
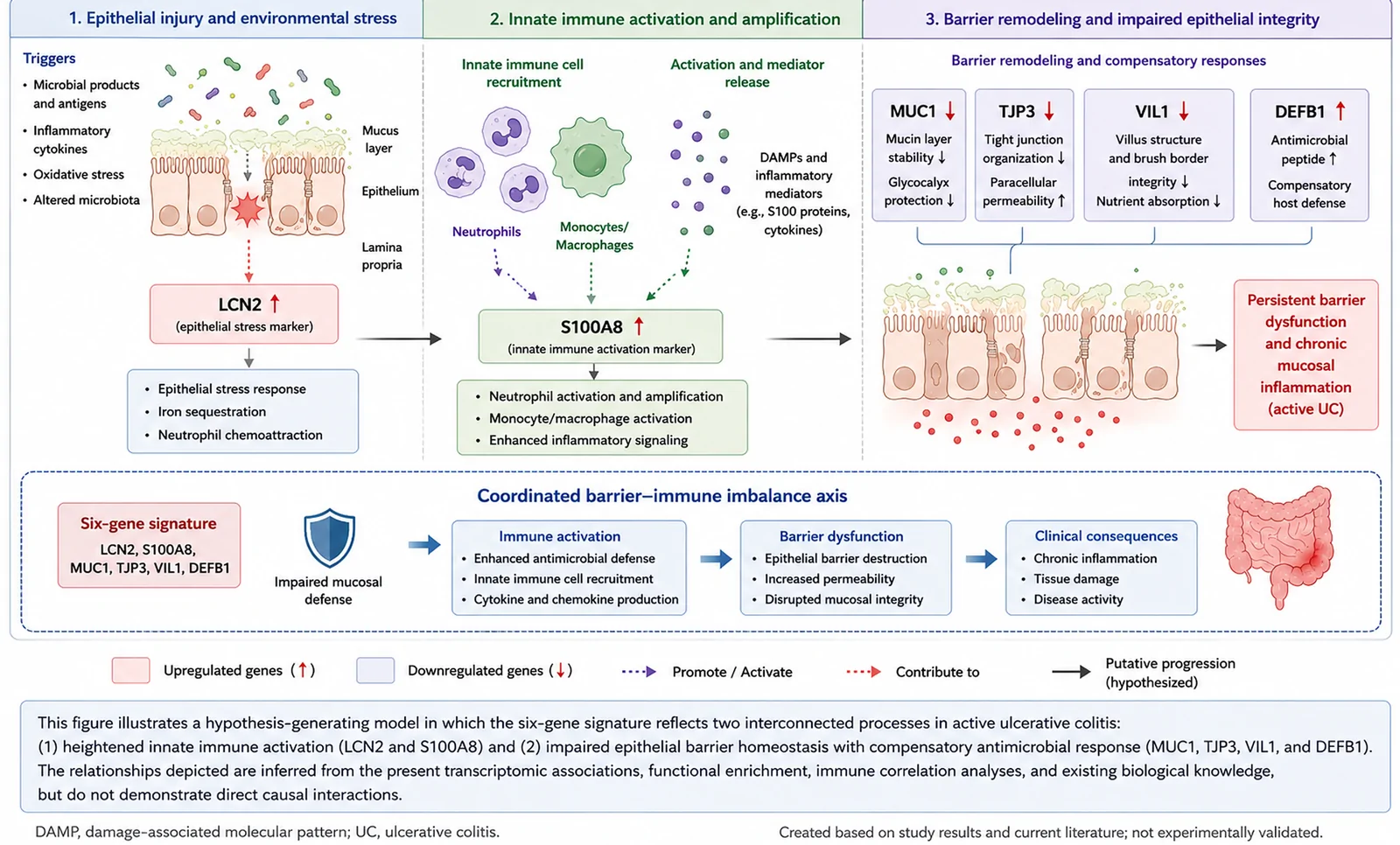
Proposed biological framework linking the six-gene transcriptomic signature to mucosal barrier injury and immune remodeling in UC. *LCN2* and *S100A8* may reflect inflammatory and innate immune activation; *MUC1*, *TJP3*, and *VIL1* may reflect remodeling of mucus, junctional, and brush-border programs; and *DEFB1* may reflect antimicrobial host defense. The relationships shown are hypotheses inferred from transcriptomic associations and previous evidence rather than experimentally demonstrated causal effects. DAMP, damage-associated molecular pattern; UC, ulcerative colitis.

The leakage-safe analyses showed that discrimination remained high after every model-fitting step was restricted to outer-training samples. The locked score achieved a mean repeated out-of-fold AUC of 0.9660, and the full fold-local feature-selection pipeline achieved 0.9600. External performance was lower in GSE38713 than in GSE92415. GSE38713 combined remission, active involved-mucosa, and active non-involved-mucosa samples, whereas GSE92415 contained samples across visits; the cohorts also differed in platform, treatment status, and sample composition. These differences plausibly contribute to the lower sensitivity and calibration observed in GSE38713. The results support cross-cohort reproducibility but do not establish clinical transportability.

Functional enrichment supported the biological interpretation of the broader transcriptomic landscape. Upregulated genes were associated with immune and defense responses, whereas downregulated genes were associated with lipid metabolism, small-molecule metabolism, and transmembrane transport. These findings indicate coexisting inflammatory activation and epithelial functional impairment in UC mucosa [16,32,33].

The central limitation is comparator scope. The model contrasts UC mucosal biopsies with healthy or non-inflammatory controls and may therefore identify inflamed, immune-rich, or barrier-damaged intestinal tissue rather than a UC-specific molecular state. It has not been tested for discrimination from Crohn's disease, infectious colitis, other inflammatory disorders, or clinically relevant differential diagnoses. Additional limitations include retrospective public datasets, heterogeneous disease activity and treatment exposure, only 11 development-cohort controls, and warnings from penalized fitting when some nested training subsets contained fewer than eight controls. The exact six-gene composition was not uniformly recovered in fold-local selection, indicating feature-selection instability despite stable discrimination. Immune analyses used marker scores rather than direct cellular measurements, and no prospective cohort or experimental validation was available. Future studies should prioritize UC versus Crohn's disease and infectious-colitis comparisons, prospective multicenter validation, assay standardization and recalibration, and gene- and protein-level biological validation.

Within these constraints, the study contributes an a priori barrier-focused and externally evaluated transcriptomic framework. Its principal value lies in integrating epithelial structure, antimicrobial defense, and innate immune activation within a reproducible modeling analysis, rather than claiming a novel clinical diagnostic assay. The lack of a significant AUC difference from *VIL1* alone further supports this cautious biological interpretation.

## 5. Conclusions

*LCN2*, *DEFB1*, *S100A8*, *MUC1*, *VIL1*, and *TJP3* form a locked public-transcriptomic score associated with UC mucosal barrier and immune remodeling. Discrimination remained high in repeated outer cross-validation and two external public cohorts, although performance varied across cohorts and the exact feature set was not uniformly regenerated by fold-local selection. The score should not be interpreted as UC-specific or clinically deployable until it is validated against relevant inflammatory disease controls in prospective cohorts.

## Supporting Information

S1 Fig Functional enrichment analysis of differentially expressed genes in GSE75214. (A) Selected significant terms for 563 upregulated genes. (B) Selected significant terms for 303 downregulated genes. Bubble size represents the intersection size and color represents the g:SCS-adjusted P value. Analyses used the 21,147 tested genes as the custom background; complete results are provided in the supporting tables archive.
(PDF)

S2 Fig Functional enrichment analysis of 24 barrier-related differentially expressed genes. Selected significant terms are shown; bubble size represents the intersection size and color represents the g:SCS-adjusted P value. The analysis used the 21,147 genes tested in GSE75214 as the custom background; complete results are provided in the supporting tables archive.
(PDF)

S3 Fig Calibration curves of the ridge regression-based six-gene transcriptomic score. (A) Calibration curve in the GSE75214 training cohort. (B) Calibration curve in the GSE38713 validation cohort. (C) Calibration curve in the GSE92415 validation cohort. The dashed line represents ideal calibration. Brier scores were calculated to quantify prediction error.
(PDF)

S1 Data Supporting Tables S1-S13. The ZIP archive contains separate Excel workbooks for Tables S1-S10 and one Excel workbook with separate worksheets for Tables S11-S13.
(ZIP)

S1 Checklist Completed TRIPOD+AI reporting checklist. (DOCX)

S1 File Complete reproducible R analysis code for the leakage-safe repeated nested-cross-validation workflow, locked six-gene ridge model, and external validation analyses. (R)

S2 File Processed expression matrix and phenotype data for the GSE75214 development cohort. (ZIP)

S3 File README describing the supporting file structure and reproduction steps. (TXT)

S4 File Processed expression matrices and phenotype data for the GSE38713 and GSE92415 validation cohorts. (ZIP)
