# Supplementary material for: A mucosal barrier injury related six gene transcriptomic signature associated with ulcerative colitis using machine learning and public transcriptomic data
Source: PLoS One. 2026 Nov 2;21(11):e0361599. doi: 10.1371/journal.pone.0361599 (PMC13726911; doi:10.1371/journal.pone.0361599)
Supplement: Supplementary file 1 [file pone.0361599.s001.pdf]

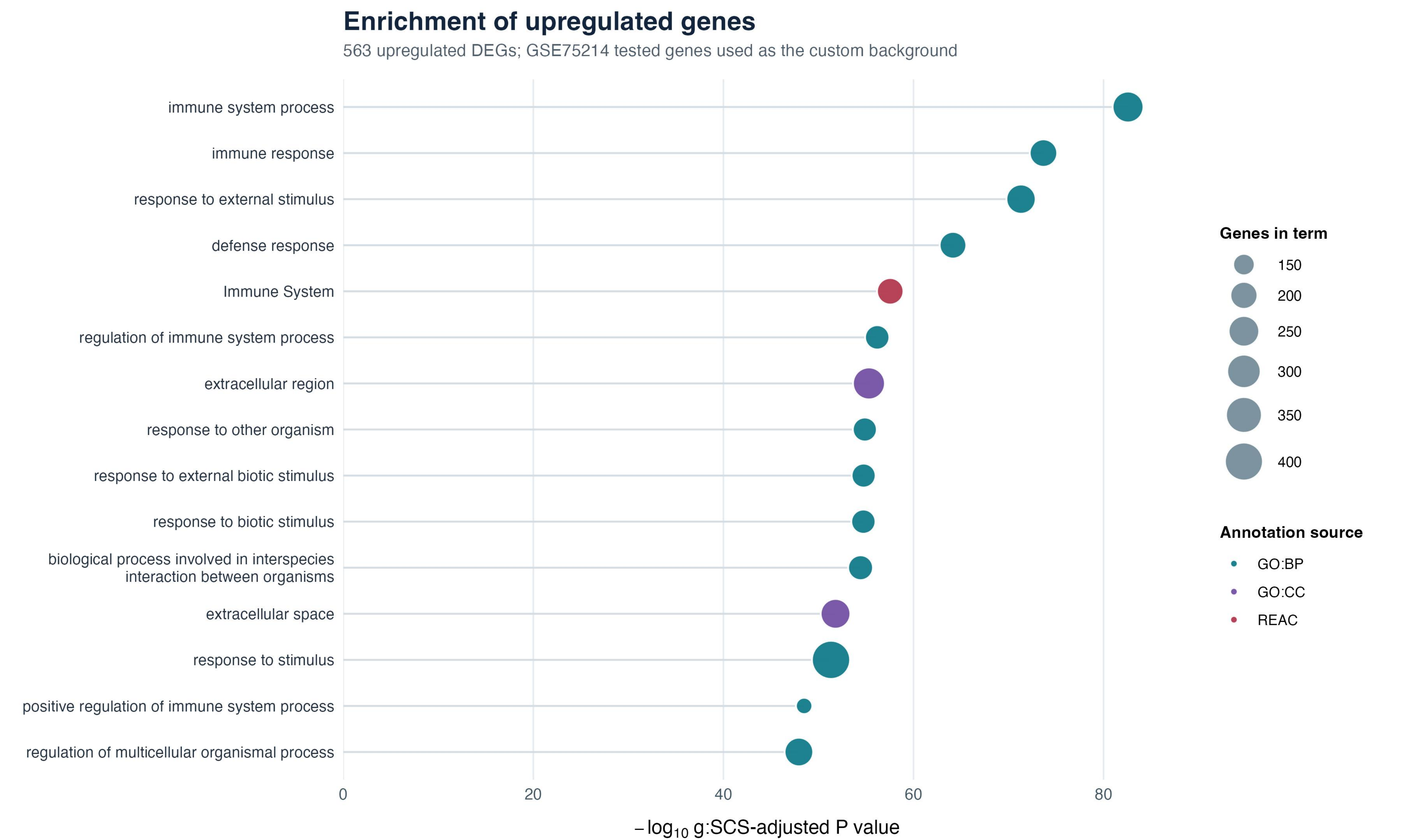
Enrichment of upregulated genes
563 upregulated DEGs; GSE75214 tested genes used as the custom background
immune system process
immune response
response to external stimulus
defense response
Immune System
regulation of immune system process
extracellular region
response to other organism
response to external biotic stimulus
response to biotic stimulus
biological process involved in interspecies interaction between organisms
extracellular space
response to stimulus
positive regulation of immune system process
regulation of multicellular organismal process
0
20
40
60
80
−log10 g:SCS-adjusted P value
Genes in term
150
200
250
300
350
400
Annotation source
GO:BP
GO:CC
REAC

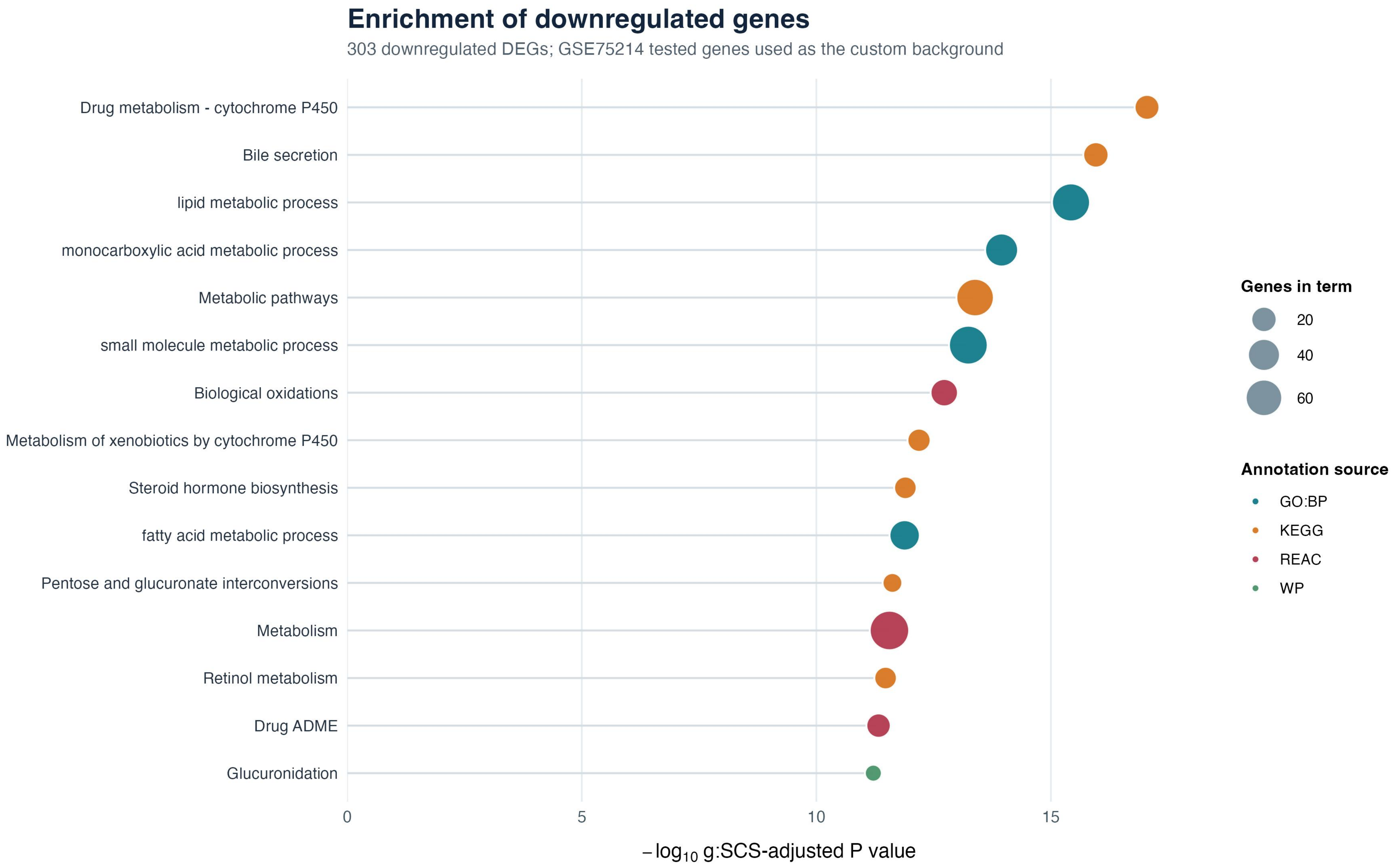
Enrichment of downregulated genes
303 downregulated DEGs; GSE75214 tested genes used as the custom background
Drug metabolism - cytochrome P450
Bile secretion
lipid metabolic process
monocarboxylic acid metabolic process
Metabolic pathways
small molecule metabolic process
Biological oxidations
Metabolism of xenobiotics by cytochrome P450
Steroid hormone biosynthesis
fatty acid metabolic process
Pentose and glucuronate interconversions
Metabolism
Retinol metabolism
Drug ADME
Glucuronidation
0
5
10
15
$-\log_{10}$ g:SCS-adjusted P value
Genes in term
20
40
60
Annotation source
GO:BP
KEGG
REAC
WP
