# Supplementary material for: A mucosal barrier injury related six gene transcriptomic signature associated with ulcerative colitis using machine learning and public transcriptomic data
Source: PLoS One. 2026 Nov 2;21(11):e0361599. doi: 10.1371/journal.pone.0361599 (PMC13726911; doi:10.1371/journal.pone.0361599)
Supplement: Supplementary file 2 [file pone.0361599.s002.pdf]

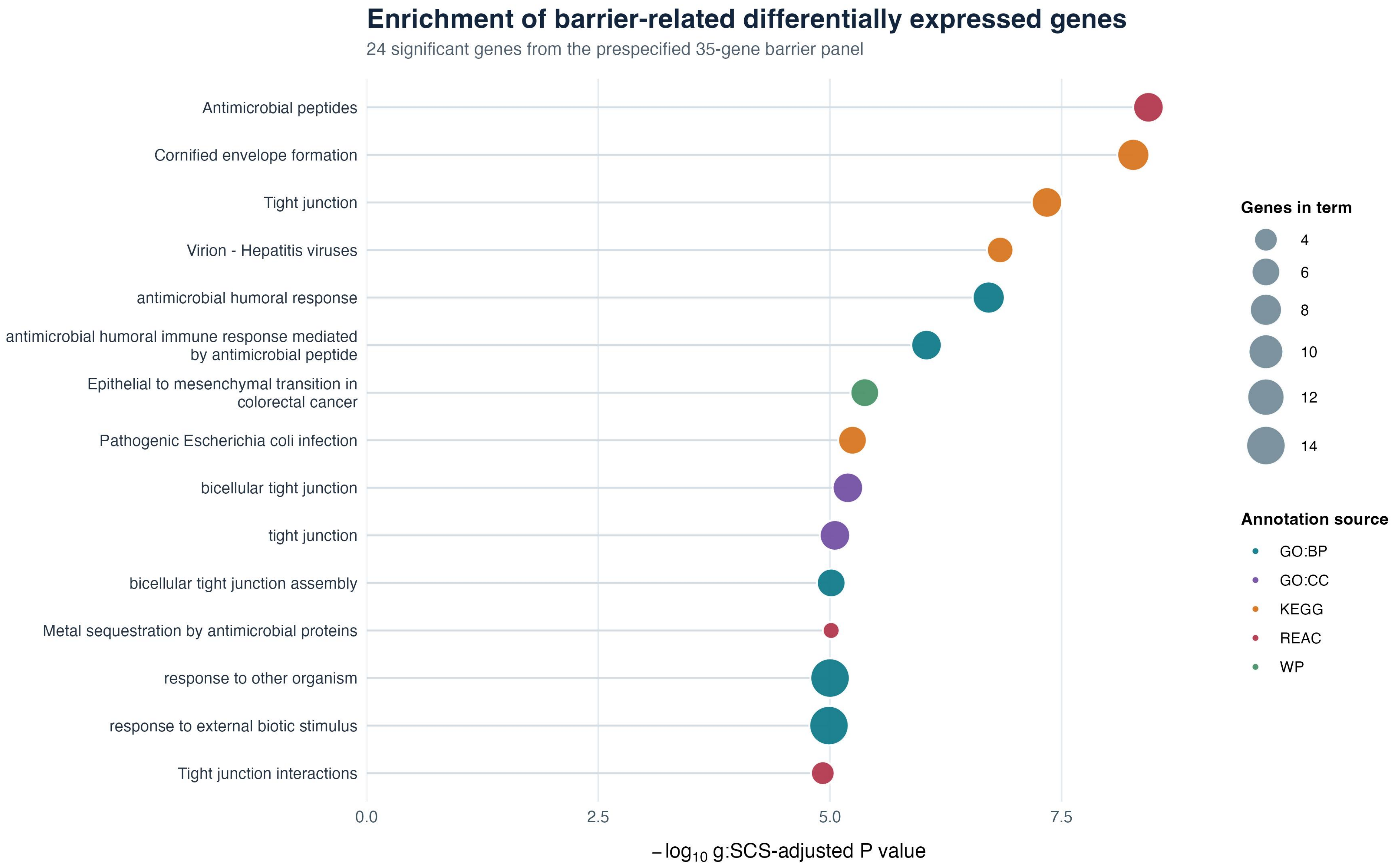
Enrichment of barrier-related differentially expressed genes
24 significant genes from the prespecified 35-gene barrier panel
Antimicrobial peptides
Cornified envelope formation
Tight junction
Virion - Hepatitis viruses
antimicrobial humoral response
antimicrobial humoral immune response mediated by antimicrobial peptide
Epithelial to mesenchymal transition in colorectal cancer
Pathogenic Escherichia coli infection
bicellular tight junction
tight junction
bicellular tight junction assembly
Metal sequestration by antimicrobial proteins
response to other organism
response to external biotic stimulus
Tight junction interactions
0.0
2.5
5.0
7.5
−log10 g:SCS-adjusted P value
Genes in term
4
6
8
10
12
14
Annotation source
GO:BP
GO:CC
KEGG
REAC
WP
