# Supplementary material for: A mucosal barrier injury related six gene transcriptomic signature associated with ulcerative colitis using machine learning and public transcriptomic data
Source: PLoS One. 2026 Nov 2;21(11):e0361599. doi: 10.1371/journal.pone.0361599 (PMC13726911; doi:10.1371/journal.pone.0361599)
Supplement: Supplementary file 3 [file pone.0361599.s003.pdf]

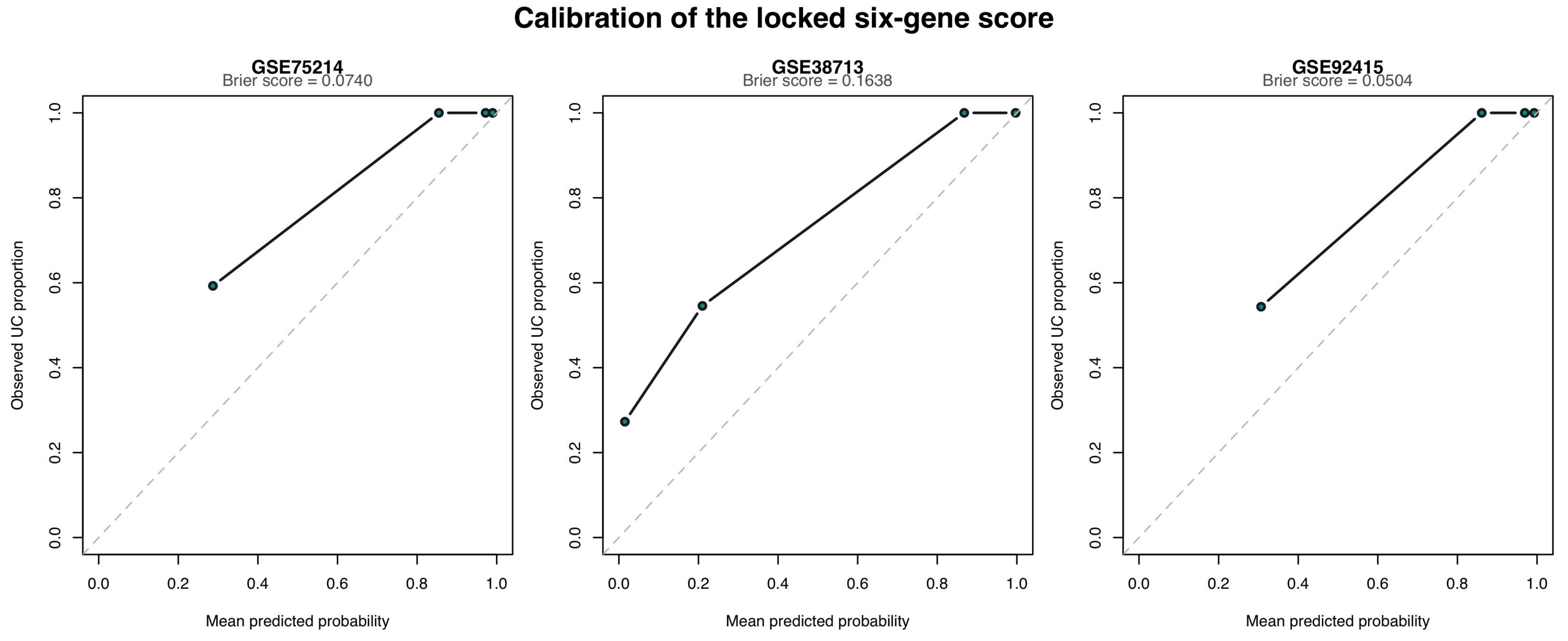

Calibration of the locked six-gene score
GSE75214
Brier score = 0.0740
GSE38713
Brier score = 0.1638
GSE92415
Brier score = 0.0504
Observed UC proportion
Mean predicted probability
0.0
0.2
0.4
0.6
0.8
1.0
