# Supplementary material for: A mucosal barrier injury related six gene transcriptomic signature associated with ulcerative colitis using machine learning and public transcriptomic data
Source: PLoS One. 2026 Nov 2;21(11):e0361599. doi: 10.1371/journal.pone.0361599 (PMC13726911; doi:10.1371/journal.pone.0361599)
Supplement: Supplementary file 5 [file pone.0361599.s005.docx]

S1 Checklist. TRIPOD+AI reporting checklist

Study: A Mucosal Barrier Injury Related Six Gene Transcriptomic Signature Associated with Ulcerative Colitis Using Machine Learning and Public Transcriptomic Data

| Section/topic | Item | Type | Checklist item (concise) | Reported on page/location |
| --- | --- | --- | --- | --- |
| Title | 1 | D;E | Identify prediction-model study, target population, and outcome | Title, p. 1 |
| Abstract | 2 | D;E | Report a structured summary of objectives, data, participants, predictors, methods, intended use, results, and conclusions | Abstract, p. 1 |
| Background | 3a | D;E | Explain healthcare context and rationale, with relevant existing models | Introduction, pp. 1–2 |
| Background | 3b | D;E | Describe target population, intended purpose, care-pathway context, and intended users | Introduction, p. 2; Discussion, pp. 6–8 |
| Background | 3c | D;E | Describe known health inequalities | Not assessable from de-identified public datasets; limitation, pp. 7–8 |
| Objectives | 4 | D;E | Specify development and/or evaluation objectives | Introduction, p. 2 |
| Data | 5a | D;E | Describe development and evaluation data sources, rationale, and representativeness | Methods 2.1, pp. 2–3; Table 1 |
| Data | 5b | D;E | Report participant accrual dates and follow-up, if applicable | Original GEO records; no longitudinal follow-up used, Methods 2.1 |
| Participants | 6a | D;E | Describe study setting, centres, and locations | Methods 2.1 and cited GEO records |
| Participants | 6b | D;E | Describe eligibility criteria | Methods 2.1, pp. 2–3 |
| Participants | 6c | D;E | Describe treatments and handling, if relevant | Treatment metadata not used; Discussion limitations, pp. 7–8 |
| Data preparation | 7 | D;E | Describe preprocessing, quality checking, harmonisation, and subgroup consistency | Methods 2.1, pp. 2–3; S1 File |
| Outcome | 8a | D;E | Define predicted outcome and assessment | Methods 2.1 and 2.6, pp. 2–4 |
| Outcome | 8b | D;E | Describe subjective outcome assessors, if applicable | Not applicable; GEO labels were used |
| Outcome | 8c | D;E | Report outcome-assessment blinding | Not applicable to retrospective GEO labels |
| Predictors | 9a | D;E | Describe initial predictor choice and preselection | Methods 2.3 and 2.5, pp. 3–4; S3/S8 Tables |
| Predictors | 9b | D;E | Define predictors and measurement | Methods 2.1, 2.3, and 2.6; S3/S9 Tables |
| Predictors | 9c | D;E | Describe subjective predictor assessors, if applicable | Not applicable; microarray expression measurements |
| Sample size | 10 | D;E | Explain development and evaluation sample sizes | All eligible public samples used; Methods 2.1; Table 1 |
| Missing data | 11 | D;E | Describe missing-data handling and omissions | Methods 2.1; complete six-gene matrices used |
| Analytical methods | 12a | D;E | Describe data partitioning and internal validation | Methods 2.6, pp. 3–4; Figure 1; S1 File |
| Analytical methods | 12b | D;E | Describe transformations and standardisation | Methods 2.1 and 2.6; S9 Table |
| Analytical methods | 12c | D;E | Specify model rationale, all building/tuning steps, and internal validation | Methods 2.5–2.6; Figures 4–5; S1 File |
| Analytical methods | 12d | D;E | Describe handling of clustered heterogeneity | Not performed; cohorts evaluated separately |
| Analytical methods | 12e | D;E | Specify discrimination, calibration, utility, and comparison measures | Methods 2.6 and 2.8; Figures 5–7; S3 Figure |
| Analytical methods | 12f | D;E | Describe model updating after evaluation | No external updating or recalibration performed |
| Analytical methods | 12g | D;E | Describe calculation of evaluation predictions | Methods 2.6; S1 File; S9 Table |
| Class imbalance | 13 | D;E | Describe class-imbalance handling and recalibration | Methods 2.5–2.6; inverse-frequency weights; no external recalibration |
| Fairness | 14 | D;E | Describe fairness approaches | Not assessable because key sociodemographic variables were unavailable; limitation |
| Model output | 15 | D;E | Specify output and classification threshold | Methods 2.6; probability score and training-derived cutoff 0.4974 |
| Training vs evaluation | 16 | D;E | Identify differences between development and evaluation data | Methods 2.1; Table 1; Discussion, pp. 7–8 |
| Ethics | 17 | D;E | Report ethics approval or waiver | Methods/Declarations, p. 20; public de-identified data |
| Open science | 18a | D;E | Funding source and funder role | Funding statement, p. 20; research unfunded, APC support disclosed |
| Open science | 18b | D;E | Conflicts of interest | Competing interests, p. 20 |
| Open science | 18c | D;E | Protocol availability | No prospective protocol was prepared; Reporting Guidelines, p. 4 |
| Open science | 18d | D;E | Registration information | Study was not registered; Reporting Guidelines, p. 4 |
| Open science | 18e | D;E | Data availability | Data Availability Statement, p. 20; S2 File |
| Open science | 18f | D;E | Code availability | Data Availability Statement; S1 File |
| PPI | 19 | D;E | Describe patient/public involvement | None; Reporting Guidelines, p. 4 |
| Participants | 20a | D;E | Report participant flow and outcome counts | Results 3.1; Figure 1; Table 1 |
| Participants | 20b | D;E | Report characteristics by data source, dates, predictors, treatments, missing data, and demographics | Table 1; S1 Table; unavailable metadata acknowledged |
| Participants | 20c | D;E | Compare important predictor distributions across development/evaluation datasets | Figures 3 and 6; cohorts were not pooled |
| Model development | 21 | D;E | Report participants and events in each analysis | Results 3.1 and 3.6; Table 1 |
| Model specification | 22 | D;E | Provide full model for third-party prediction | S1 File and S9 Table |
| Model performance | 23a | D;E | Report performance with confidence intervals and plots | Results 3.6; Tables 2–3; Figures 5–7; S3 Figure |
| Model performance | 23b | D;E | Report cluster heterogeneity if examined | Not examined; datasets analyzed separately |
| Model updating | 24 | D;E | Report model updating after evaluation | Not applicable; locked model was not updated |
| Interpretation | 25 | D;E | Interpret results in context, including fairness | Discussion, pp. 6–8 |
| Limitations | 26 | D;E | Discuss bias, uncertainty, overfitting, data quality, and generalisability | Discussion, pp. 7–8 |
| Usability | 27a | D;E | Discuss poor-quality or unavailable inputs at implementation | Not ready for implementation; future standardisation required, Discussion |
| Usability | 27b | D;E | Describe user interaction and expertise | Not applicable at this hypothesis-generating stage |
| Usability | 27c | D;E | Describe next research steps for applicability and generalisability | Discussion and Conclusions, pp. 7–8 |

Source: Collins GS, Moons KGM, Dhiman P, et al. TRIPOD+AI statement. BMJ. 2024;385:e078378. Checklist version 11 January 2024.
