# Supplementary material for: A mucosal barrier injury related six gene transcriptomic signature associated with ulcerative colitis using machine learning and public transcriptomic data
Source: PLoS One. 2026 Nov 2;21(11):e0361599. doi: 10.1371/journal.pone.0361599 (PMC13726911; doi:10.1371/journal.pone.0361599)
Supplement: Supplementary file 8 [file pone.0361599.s011.docx]

Response to the Academic Editor and Reviewers

Manuscript: A Mucosal Barrier Injury Related Six Gene Transcriptomic Signature Associated with Ulcerative Colitis Using Machine Learning and Public Transcriptomic Data

Dear Academic Editor and Reviewers,

We sincerely thank you for the careful and constructive evaluation. We extensively revised the manuscript, repeated the internal validation using leakage-safe nested cross-validation, corrected and expanded the methods and reporting, regenerated figures and supporting tables, and provided complete code and processed data. Changes are highlighted in the marked manuscript. Our principal revisions are summarized below.

- Reframed the work as a candidate transcriptomic signature associated with UC mucosal barrier–immune imbalance, not a UC-specific clinical diagnostic assay.
- Repeated 50× stratified five-fold outer cross-validation; a sensitivity analysis repeated DEG filtering, LASSO, random-forest prioritization, and ridge fitting within each outer-training fold.
- Applied a locked training-derived model and cutoff to both external cohorts; corrected GSE38713 AUC to 0.8974 (95% CI 0.8040–0.9909).
- Compared VIL1 with the six-gene model using DeLong’s test (P=0.4026) and removed any claim of significant incremental discrimination.
- Expanded dataset selection, preprocessing, barrier-panel derivation, feature-selection, limitations, legends, and Supporting Information.

# Academic Editor and journal requirements

## Editor 1–4: style, ORCID, code, and data availability

**Comment:** Please meet PLOS ONE style requirements, validate the corresponding author ORCID, share author-generated code, and provide a complete Data Availability Statement.

**Response:** The manuscript and filenames were standardized. Complete R code (S1 File), processed expression/phenotype data (S2 File), and a README (S3 File) are supplied. The Data Availability Statement identifies all GEO accessions and supporting files. The corresponding author will verify ORCID in Editorial Manager before submission.

## Editor 5: funding statement

**Comment:** Funding information in the manuscript conflicts with the online statement. Remove funding information from Acknowledgments and provide an amended statement in the cover letter.

**Response:** We clarified that the research itself received no specific funding and that the National Natural Science Foundation of China [grant number 82560937] supported only the article processing charge. Funding information appears only in the dedicated Funding section, not in Acknowledgments. We request that the online Funding Statement be amended to: “The authors received no specific funding for the research reported in this article. The article processing charge was supported by the National Natural Science Foundation of China [grant number 82560937]. The funder had no role in study design, data collection and analysis, preparation of the manuscript, or the decision to submit the article for publication.”

## Editor 6–8: Figure 10, Table 1, and separate Supporting Information

**Comment:** Upload Figure 10 and Table 1, and remove supporting figures/tables from the manuscript body for separate upload.

**Response:** Figure 10 is supplied as a separate high-resolution file, Table 1 remains in the manuscript, and all supporting figures and tables are supplied as separate Supporting Information files. Their captions are listed after the main figure legends.

## Editor 9: copyrighted material in Figures 1 and 2

**Comment:** Provide permission or replacement figures compatible with CC BY 4.0.

**Response:** Figures 1 and 2 were completely redrawn by the authors from the study workflow and numerical results. The replacements contain no copied third-party artwork and are suitable for publication under CC BY 4.0.

## Editor 10–11: captions and missing Supporting Information

**Comment:** Provide Supporting Information captions after the references and upload S3, S4, S9, S11, S12, and S13 Tables.

**Response:** The manuscript now contains a complete Additional Files checklist and captions. S1–S10 Tables are separate workbooks; S11–S13 Tables are separate worksheets in a single workbook. All specifically requested tables are included.

# Reviewer #1

## Reviewer #1, comment 1

**Comment:** Introduction: novelty and limitations of existing signatures

**Response:** We now state that many previous panels were optimized primarily for case-control discrimination and offered limited a priori biological organization. We clarify that our contribution is the prespecified barrier-focused framework and coordinated representation of innate immune activation, antimicrobial defense, epithelial structure, and junctional integrity—not discovery of previously unknown UC genes.

## Reviewer #1, comment 2

**Comment:** Methods: derivation of the 35 barrier-related genes

**Response:** Section 2.3 now describes the literature and functional categories, search terms, retention logic, symbol harmonization, duplicate handling, detectability check, and the final prespecified set. The complete panel and categories are provided in S3 Table.

## Reviewer #1, comment 3

**Comment:** Methods: normalization, harmonization, and batch effects

**Response:** Section 2.1 now reports series-matrix processing, probe-to-gene mapping and averaging, transformations/normalization checks, and independent cohort processing. The cohorts were never merged; therefore cross-cohort ComBat correction was not applied. External validation instead used training-derived scaling and a locked model.

## Reviewer #1, comment 4

**Comment:** Results: selection rules, DEFA5, and class imbalance

**Response:** We explicitly report that DEFA5 passed differential-expression screening but was not retained by the intersection of the LASSO and random-forest priority sets. Inverse-frequency class weights were used during model fitting, and stratification was used for all folds.

## Reviewer #1, comment 5

**Comment:** Discussion: lower GSE38713 performance and generalizability

**Response:** The Discussion now explains that the lower AUC in GSE38713 may reflect platform, sample-composition, disease-activity, biopsy-location, and cohort-size differences. We emphasize the need for prospective, assay-standardized validation against Crohn’s disease, infectious colitis, and other inflammatory controls.

# Reviewer #2 — Major concerns

## Reviewer #2, major comment 1

**Comment:** The term “diagnostic signature” may overstate clinical scope.

**Response:** We agree. “Diagnostic” was removed from the title and the model is consistently described as a locked public-transcriptomic score/candidate molecular signature. The Abstract, Introduction, Discussion, and Conclusions now emphasize that it has not established UC specificity or clinical readiness.

## Reviewer #2, major comment 2

**Comment:** Clarify the additional biological insight beyond existing signatures.

**Response:** We no longer claim novel gene discovery. The revised text identifies the contribution as a biologically prespecified framework linking inflammation-associated genes (LCN2/S100A8), antimicrobial defense (DEFB1), mucin/junctional biology (MUC1/TJP3), and epithelial differentiation (VIL1), supported by enrichment and immune-marker correlations.

## Reviewer #2, major comment 3

**Comment:** Verify citations and distinguish the present work from genuinely IBD/UC-focused studies.

**Response: We verified all cited references. The GEO database paper was removed from the sentence describing prior UC machine-learning studies, the atrial-fibrillation and atopic-dermatitis articles were deleted, and UC-specific primary studies were cited instead. We also revised the Introduction to distinguish our prespecified 35-gene barrier panel, leakage-aware internal validation, and locked fixed-cutoff external evaluation from prior data-driven or broad-panel approaches. We now explicitly state that the contribution is the barrier-focused analytical organization and validation framework rather than discovery of previously unknown UC genes.**

## Reviewer #2, major comment 4

**Comment:** The model may detect nonspecific inflamed, damaged tissue rather than UC.

**Response:** We agree and elevated this from a secondary limitation to a central caveat throughout the manuscript. The score is interpreted as a pattern associated with UC mucosal inflammation and barrier injury; future evaluation against Crohn’s disease, infectious colitis, and non-IBD inflammatory controls is explicitly required.

## Reviewer #2, major comment 5

**Comment:** Potential information leakage; repeat modeling with nested cross-validation if necessary.

**Response:** Audit of the original code confirmed that feature selection occurred on the full GSE75214 cohort before cross-validation of the final model. We therefore added 50 repetitions of stratified five-fold outer cross-validation. In the primary locked-score analysis, preprocessing and ridge tuning were confined to each outer-training fold (mean out-of-fold AUC 0.9660, SD 0.0031). In a stricter sensitivity analysis, differential-expression filtering, LASSO, random-forest ranking, and ridge fitting were all repeated within each outer-training fold (mean AUC 0.9600, SD 0.0094). These results and complete code/predictions are supplied.

# Reviewer #2 — Minor comments

## Reviewer #2, minor comment 1

**Comment:** Italicize gene symbols throughout.

**Response:** Gene symbols were standardized and italicized in narrative text and figure labels where typographically appropriate.

## Reviewer #2, minor comment 2

**Comment:** Correct the GSE38713 AUC discrepancy.

**Response:** All analyses and displays now report AUC 0.8974 (95% CI 0.8040–0.9909) from the locked model.

## Reviewer #2, minor comment 3

**Comment:** Provide higher-resolution Figures 1 and 10.

**Response:** Both figures were regenerated/provided at publication-quality resolution.

## Reviewer #2, minor comment 4

**Comment:** Test incremental value over VIL1 alone.

**Response:** A paired DeLong comparison was added. The six-gene AUC (0.9747) was not significantly higher than VIL1 alone (0.9616; P=0.4026). We therefore frame the six-gene score as broader biological representation rather than claiming statistically superior discrimination.

## Reviewer #2, minor comment 5

**Comment:** Verify Figure 7/8 placement and duplication.

**Response:** The figure set was audited. Figure 7 contains decision-curve analysis; Figure 8 contains immune marker-score heatmap and boxplots. Duplicate/misplaced files were removed from the submission set.

## Reviewer #2, minor comment 6

**Comment:** Remove dashes/underscores in immune-cell names.

**Response:** Labels were standardized, including “CD4+ T cells” and “CD8+ T cells”; Figures 8A, 8B, and 9 were regenerated.

We again thank the editor and reviewers. We believe the revised manuscript is more transparent, reproducible, and appropriately calibrated to the evidence.

Sincerely,
The Authors
