# Supplementary material for: A mucosal barrier injury related six gene transcriptomic signature associated with ulcerative colitis using machine learning and public transcriptomic data
Source: PLoS One. 2026 Nov 2;21(11):e0361599. doi: 10.1371/journal.pone.0361599 (PMC13726911; doi:10.1371/journal.pone.0361599)
Supplement: Peer review history

# Peer review: Decision Letter 0

**DOI:** 10.1371/journal.pone.0361599.r001

**Authors:** Yash Gupta

13 Sep 2026

Dear Dr. he,

Thank you for submitting your manuscript to PLOS One. After careful consideration, we feel that it has merit but does not fully meet PLOS One’s publication criteria as it currently stands. Therefore, we invite you to submit a revised version of the manuscript that addresses the points raised during the review process.

Please submit your revised manuscript by Nov 12 2026 11:59PM. If you will need more time than this to complete your revisions, please reply to this message or contact the journal office at plosone@plos.org. When you're ready to submit your revision, log on to https://www.editorialmanager.com/pone/ and select the 'Submissions Needing Revision' folder to locate your manuscript file.

- A letter that responds to each point raised by the academic editor and reviewer(s). You should upload this letter as a separate file labeled 'Response to Reviewers'.
- A marked-up copy of your manuscript that highlights changes made to the original version. You should upload this as a separate file labeled 'Revised Manuscript with Track Changes'.
- An unmarked version of your revised paper without tracked changes. You should upload this as a separate file labeled 'Manuscript'.

If you would like to make changes to your financial disclosure, please include your updated statement in your cover letter. Guidelines for resubmitting your figure files are available below the reviewer comments at the end of this letter.

If applicable, we recommend that you deposit your laboratory protocols in protocols.io to enhance the reproducibility of your results. Protocols.io assigns your protocol its own identifier (DOI) so that it can be cited independently in the future. For instructions see: https://journals.plos.org/plosone/s/submission-guidelines#loc-laboratory-protocols. Additionally, PLOS One offers an option for publishing peer-reviewed Lab Protocol articles, which describe protocols hosted on protocols.io. Read more information on sharing protocols at https://plos.org/protocols?utm_medium=editorial-email&utm_source=authorletters&utm_campaign=protocols.

As the corresponding author, your ORCID iD is verified in the submission system and will appear in the published article. PLOS supports the use of ORCID, and we encourage all coauthors to register for an ORCID iD and use it as well. Please encourage your coauthors to verify their ORCID iD within the submission system before final acceptance, as unverified ORCID iDs will not appear in the published article. *Only*  the individual author can complete the verification step; PLOS staff *cannot*  verify ORCID iDs on behalf of authors.

We look forward to receiving your revised manuscript.

Kind regards,

Yash Gupta, Ph.D.

Academic Editor

PLOS One

Journal Requirements:

When submitting your revision, we need you to address these additional requirements.

1. Please ensure that your manuscript meets PLOS ONE's style requirements, including those for file naming. The PLOS ONE style templates can be found at

https://journals.plos.org/plosone/s/file?id=wjVg/PLOSOne_formatting_sample_main_body.pdf and https://journals.plos.org/plosone/s/file?id=ba62/PLOSOne_formatting_sample_title_authors_affiliations.pdf

2. PLOS requires an ORCID iD for the corresponding author in Editorial Manager on papers submitted after December 6th, 2016. Please ensure that you have an ORCID iD and that it is validated in Editorial Manager. To do this, go to ‘Update my Information’ (in the upper left-hand corner of the main menu), and click on the Fetch/Validate link next to the ORCID field. This will take you to the ORCID site and allow you to create a new iD or authenticate a pre-existing iD in Editorial Manager.

3. Please note that PLOS One has specific guidelines on code sharing for submissions in which author-generated code underpins the findings in the manuscript. In these cases, we expect all author-generated code to be made available without restrictions upon publication of the work. Please review our guidelines at https://journals.plos.org/plosone/s/materials-and-software-sharing#loc-sharing-code and ensure that your code is shared in a way that follows best practice and facilitates reproducibility and reuse.

4. Please provide a complete Data Availability Statement in the submission form, ensuring you include all necessary access information or a reason for why you are unable to make your data freely accessible. If your research concerns only data provided within your submission, please write "All data are in the manuscript and/or supporting information files" as your Data Availability Statement.

5. Thank you for stating the following in the Acknowledgments Section of your manuscript:

The authors thank the GEO database and the original contributors of GSE75214, GSE38713, and GSE92415 for making their transcriptomic datasets publicly available.

We note that you have provided funding information that is not currently declared in your Funding Statement. However, funding information should not appear in the Acknowledgments section or other areas of your manuscript. We will only publish funding information present in the Funding Statement section of the online submission form.

Please remove any funding-related text from the manuscript and let us know how you would like to update your Funding Statement. Currently, your Funding Statement reads as follows:

The author(s) received no specific funding for this work.

Please include your amended statements within your cover letter; we will change the online submission form on your behalf.

6. Please upload a copy of Figure 10, to which you refer in your text on page 15. If the figure is no longer to be included as part of the submission please remove all reference to it within the text.

7. Please include a copy of Table 1 which you refer to in your text on page 8.

8. We notice that your supplementary [figures/tables] are included in the manuscript file. Please remove them and upload them with the file type 'Supporting Information'. Please ensure that each Supporting Information file has a legend listed in the manuscript after the references list.

9. We note that Figure(s) 1, 2 in your submission contain copyrighted images. All PLOS content is published under the Creative Commons Attribution License (CC BY 4.0), which means that the manuscript, images, and Supporting Information files will be freely available online, and any third party is permitted to access, download, copy, distribute, and use these materials in any way, even commercially, with proper attribution. For more information, see our copyright guidelines: http://journals.plos.org/plosone/s/licenses-and-copyright.

We require you to either (1) present written permission from the copyright holder to publish these figures specifically under the CC BY 4.0 license, or (2) remove the figures from your submission:

a. You may seek permission from the original copyright holder of Figure(s) 1, 2 to publish the content specifically under the CC BY 4.0 license.

We recommend that you contact the original copyright holder with the Content Permission Form (http://journals.plos.org/plosone/s/file?id=7c09/content-permission-form.pdf) and the following text:

“I request permission for the open-access journal PLOS ONE to publish XXX under the Creative Commons Attribution License (CCAL) CC BY 4.0 (http://creativecommons.org/licenses/by/4.0/). Please be aware that this license allows unrestricted use and distribution, even commercially, by third parties. Please reply and provide explicit written permission to publish XXX under a CC BY license and complete the attached form.”

Please upload the completed Content Permission Form or other proof of granted permissions as an "Other" file with your submission.

In the figure caption of the copyrighted figure, please include the following text: “Reprinted from [ref] under a CC BY license, with permission from [name of publisher], original copyright [original copyright year].”

b. If you are unable to obtain permission from the original copyright holder to publish these figures under the CC BY 4.0 license or if the copyright holder’s requirements are incompatible with the CC BY 4.0 license, please either i) remove the figure or ii) supply a replacement figure that complies with the CC BY 4.0 license. Please check copyright information on all replacement figures and update the figure caption with source information. If applicable, please specify in the figure caption text when a figure is similar but not identical to the original image and is therefore for illustrative purposes only.

10. Please include captions for your Supporting Information files at the end of your manuscript, and update any in-text citations to match accordingly. Please see our Supporting Information guidelines for more information: http://journals.plos.org/plosone/s/supporting-information.

11. Please upload a copy of Supporting Information Table, S3, S4 S9, S11, S12, S13 which you refer to in your text on page 2, 3, 4, 5.

12. If the reviewer comments include a recommendation to cite specific previously published works, please review and evaluate these publications to determine whether they are relevant and should be cited. There is no requirement to cite these works unless the editor has indicated otherwise.

Additional Editor Comments :

Authors need to revise the manuscript and figures extensively as per reviewers guidelines. While there is no new experimentations suggested by either reviewers, there is a need for an expansion in method and sample selection criteria. This amounts to a major revision. Authors need to pay special attention to scientific conventions for gene names and figure legend description lengths as they are too compact and need frequent cross referencing to the methods and results sections.

[Note: HTML markup is below. Please do not edit.]

Reviewers' comments:

Reviewer's Responses to Questions

**Comments to the Author**

1. Is the manuscript technically sound, and do the data support the conclusions?

Reviewer #1: Yes

Reviewer #2: Partly

**********

2. Has the statistical analysis been performed appropriately and rigorously?

Reviewer #1: Yes

Reviewer #2: No

**********

3. Have the authors made all data underlying the findings in their manuscript fully available?

The PLOS Data policy

Reviewer #1: Yes

Reviewer #2: Yes

**********

4. Is the manuscript presented in an intelligible fashion and written in standard English?

Reviewer #1: Yes

Reviewer #2: Yes

**********

Reviewer #1: Introduction:

- Clearly describe the limitations of existing UC transcriptomic signatures and explain how the proposed barrier-focused signature addresses these limitations to better establish the study's novelty.

- Expand the rationale for prioritizing barrier-related genes, including why they may provide additional biological insight beyond conventional transcriptomic classifiers.

Methods:

-Describe the process used to identify, screen, and retain the 35 barrier-related genes in greater detail to improve reproducibility.

-Provide details on dataset normalization, harmonization, and batch-effect assessment to support the comparability of the GEO cohorts.

Results:

-Clarify the decision rules used for feature selection, including the exclusion of DEFA5 and the approach used to manage class imbalance, to improve transparency of model development.

Discussion:

-Expand the discussion of the lower performance observed in GSE38713 and explain how it may affect model generalizability, while outlining key steps needed for future clinical and experimental validation.

Reviewer #2: The manuscript entitled “Development and External Validation of a Mucosal Barrier Injury-Related Six-Gene Diagnostic Signature for Ulcerative Colitis Based on Machine Learning and Public Transcriptomic Data” describes the development of a six-gene transcriptomic signature for active ulcerative colitis (UC) using publicly available GEO datasets and a combination of differential expression analysis, LASSO regression, random forest feature prioritization, and ridge logistic regression. The authors identify LCN2, DEFB1, S100A8, MUC1, VIL1, and TJP3 as the final features and evaluate the resulting score in two independent public cohorts.

The manuscript addresses a relevant topic, and the use of a biologically prespecified mucosal barrier-related gene set is a strength of the study. The inclusion of independent public-cohort validation also supports the robustness of the findings. However, the overall novelty appears to be moderate, as the individual genes included in the signature have previously been implicated in UC and inflammatory bowel disease, and similar transcriptomic and machine-learning approaches have been reported. Thus, the primary contribution of the study appears to be the specific combination of these genes and the barrier-focused analytical framework, rather than the identification of novel UC biomarkers. Overall, the study has potential value, but several aspects of the manuscript would benefit from further clarification and strengthening before publication. I recommend major revision.

Strengths:

1. A major strength of this study is its deliberate integration of biological rationale into the transcriptomic feature-selection strategy, rather than relying exclusively on data-driven optimization. The authors define a priori mucosal barrier injury gene panel before performing differential expression and machine-learning analyses.

2. The final six-gene signature encompasses several biologically relevant components of intestinal inflammation and epithelial biology, including innate immune and inflammatory responses, epithelial structure and barrier-associated processes and anti-microbial host defense. This biological organization makes the signature relatively accessible from both a mechanistic and translational perspective compared with purely data-driven gene panels.

3. The analytical workflow is comprehensive and generally well-structured. The use of both LASSO and random forest provides complementary approaches for feature prioritization, while ridge logistic regression is appropriate for constructing a multigene score while retaining correlated predictors. The use of stratified cross-validation and class weighting appropriately addresses the imbalanced training cohort. A further strength is the application of the training-derived cutoff (0.4974) directly to both validation cohorts rather than optimizing separate cutoffs, providing a more stringent assessment of model transportability. Finally, validation in two independent cohorts, with different sample sizes, strengthens confidence that the signature is not entirely dependent on the training dataset.

4. The authors consistently and explicitly frame the signature as a "candidate molecular framework" and "hypothesis-generating" tool rather than a ready-to-use clinical assay. This is the correct framing for a retrospective public-cohort study and is stated clearly in the abstract, introduction, results, and discussion.

Major concerns:

1. The term “diagnostic signature” in the title may overstate the clinical scope of the current analysis. Although the six-gene model effectively distinguishes active UC from healthy controls, this does not establish UC specificity or demonstrate that it specifically reflects mucosal barrier integrity.

2. In the introduction, the authors state that previous UC signatures provide limited interpretation of mucosal biology, but it is unclear what new biological insight the present study provides beyond this limitation. The selection of a barrier-focused gene set may improve biological framing but does not by itself demonstrate a novel biological finding. The authors should clarify what additional biological information or insight their six-gene signature provides compared with existing UC signatures.

3. The statement that several machine-learning studies have explored transcriptomic markers for IBD and UC [11,12,31–33] should be clarified. Notably, two of the cited references are not related to IBD/UC, and therefore do not directly support this statement. The authors should verify that the cited references are appropriate and directly relevant and should clarify how the genuinely IBD/UC-focused studies differ from the present work. If these references are used to establish the novelty of the barrier-focused approach, the authors should explain what new biological or methodological information their study provides beyond existing IBD/UC transcriptomic signatures.

4. The model distinguishes active UC from healthy colon, but it does not necessarily distinguish UC from other inflammatory diseases. Several of the signature genes (e.g., genes encoding LCN2, S100A8) are well-established non-specific inflammation markers that are expected to be elevated in many intestinal inflammatory conditions. The model is essentially detecting “inflamed, immune-rich, damaged intestinal tissues” rather than “this tissue has active ulcerative colitis”. The authors acknowledge this limitation but understate its clinical significance. A diagnostic signature that cannot differentiate active UC from Crohn’s disease or infectious colitis has severely constrained translational value. This should be elevated from a limitation to a central caveat, foregrounded much earlier in the manuscript.

5. With 11 controls in the training dataset, the AUC for Vil1 and six-gene model is unusually strong. The external validation, however, makes the concern less severe. In the current description, differential-expression filtering, LASSO selection and random-forest prioritization appear to have been performed on the full training cohort before the subsequent 5-fold cross-validation of the final model. This may result in information leakage and optimistic estimates of internal model performance. The authors should clarify the exact order of these steps and, if necessary, repeat the modeling using nested cross-validation in which all data-dependent feature-selection steps are performed within each training fold.

Minor comments:

1. Gene names should be italicized throughout the text (e.g., abstract, Fig. 3B, Fig 4A, 4B).

2. The abstract and Table 3 report the GSE38713 AUC as 0.8897, but the ROC curve in Figure 6A displays "AUC = 0.85." Authors need to fix the discrepancy.

3. The authors should provide a higher-resolution version of Figure 1 and 10, as the current figures are blurry and difficult to read.

4. The authors provide a rationale for selecting the six-gene signature based on their feature-selection strategy and biological considerations. The authors should clarify whether the six-gene combination provide demonstrable incremental predictive value over VIL1 alone? VIL1 already has an AUC of 0.9616 which is very close to the six-gene model’s 0.9747. A formal comparison of ROC curves/AUCs (e.g., DeLong’s test) could help establish the added value of the combined signature.

5. In the submitted manuscript, the immune cell marker score heatmap and boxplot panels appear to be represented twice in the extracted figure content, once in association with Figure 7 and again with Figure 8. However, the figure legends indicate that Figure 7 should present the decision curve analyses, whereas Figure 8 should present the immune marker score analysis. The authors should verify that the correct figure panels are linked to each figure number and that no figures have been inadvertently duplicated or misplaced.

6. In figures 7A, 8A-B, eliminate the dashes/underscores from the immune cell names. For example, CD4_T_cells should be changed to CD4+ T cells.

**********

what does this mean?). If published, this will include your full peer review and any attached files.

If you choose “no”, your identity will remain anonymous but your review may still be made public.

**Do you want your identity to be public for this peer review?**  For information about this choice, including consent withdrawal, please see our Privacy Policy

Reviewer #1: **Yes:**  Sandeep K. Meharwal

Reviewer #2: No

**********

[NOTE: If reviewer comments were submitted as an attachment file, they will be attached to this email and accessible via the submission site. Please log into your account, locate the manuscript record, and check for the action link "View Attachments". If this link does not appear, there are no attachment files.]

To ensure your figures meet our technical requirements, please review our figure guidelines: https://journals.plos.org/plosone/s/figures

You may also use PLOS’s free figure tool, NAAS, to help you prepare publication quality figures: https://journals.plos.org/plosone/s/figures#loc-tools-for-figure-preparation.

NAAS will assess whether your figures meet our technical requirements by comparing each figure against our figure specifications.

# Author comment: Author response to Decision Letter 1

**DOI:** 10.1371/journal.pone.0361599.r002

16 Sep 2026

Manuscript: A Mucosal Barrier Injury Related Six Gene Transcriptomic Signature Associated with Ulcerative Colitis Using Machine Learning and Public Transcriptomic Data

Dear Academic Editor and Reviewers,

We sincerely thank you for the careful and constructive evaluation. We extensively revised the manuscript, repeated the internal validation using leakage-safe nested cross-validation, corrected and expanded the methods and reporting, regenerated figures and supporting tables, and provided complete code and processed data. Changes are highlighted in the marked manuscript. Our principal revisions are summarized below.

�Reframed the work as a candidate transcriptomic signature associated with UC mucosal barrier–immune imbalance, not a UC-specific clinical diagnostic assay.

�Repeated 50× stratified five-fold outer cross-validation; a sensitivity analysis repeated DEG filtering, LASSO, random-forest prioritization, and ridge fitting within each outer-training fold.

�Applied a locked training-derived model and cutoff to both external cohorts; corrected GSE38713 AUC to 0.8974 (95% CI 0.8040–0.9909).

�Compared VIL1 with the six-gene model using DeLong’s test (P=0.4026) and removed any claim of significant incremental discrimination.

�Expanded dataset selection, preprocessing, barrier-panel derivation, feature-selection, limitations, legends, and Supporting Information.

Academic Editor and journal requirements

Editor 1–4: style, ORCID, code, and data availability

Comment: Please meet PLOS ONE style requirements, validate the corresponding author ORCID, share author-generated code, and provide a complete Data Availability Statement.

Response: The manuscript and filenames were standardized. Complete R code (S1 File), processed expression/phenotype data (S2 File), and a README (S3 File) are supplied. The Data Availability Statement identifies all GEO accessions and supporting files. The corresponding author will verify ORCID in Editorial Manager before submission.

Editor 5: funding statement

Comment: Funding information in the manuscript conflicts with the online statement. Remove funding information from Acknowledgments and provide an amended statement in the cover letter.

Response: We clarified that the research itself received no specific funding and that the National Natural Science Foundation of China [grant number 82560937] supported only the article processing charge. Funding information appears only in the dedicated Funding section, not in Acknowledgments. We request that the online Funding Statement be amended to: “The authors received no specific funding for the research reported in this article. The article processing charge was supported by the National Natural Science Foundation of China [grant number 82560937]. The funder had no role in study design, data collection and analysis, preparation of the manuscript, or the decision to submit the article for publication.”

Editor 6–8: Figure 10, Table 1, and separate Supporting Information

Comment: Upload Figure 10 and Table 1, and remove supporting figures/tables from the manuscript body for separate upload.

Response: Figure 10 is supplied as a separate high-resolution file, Table 1 remains in the manuscript, and all supporting figures and tables are supplied as separate Supporting Information files. Their captions are listed after the main figure legends.

Editor 9: copyrighted material in Figures 1 and 2

Comment: Provide permission or replacement figures compatible with CC BY 4.0.

Response: Figures 1 and 2 were completely redrawn by the authors from the study workflow and numerical results. The replacements contain no copied third-party artwork and are suitable for publication under CC BY 4.0.

Editor 10–11: captions and missing Supporting Information

Comment: Provide Supporting Information captions after the references and upload S3, S4, S9, S11, S12, and S13 Tables.

Response: The manuscript now contains a complete Additional Files checklist and captions. S1–S10 Tables are separate workbooks; S11–S13 Tables are separate worksheets in a single workbook. All specifically requested tables are included.

Reviewer #1

Reviewer #1, comment 1

Comment: Introduction: novelty and limitations of existing signatures

Response: We now state that many previous panels were optimized primarily for case-control discrimination and offered limited a priori biological organization. We clarify that our contribution is the prespecified barrier-focused framework and coordinated representation of innate immune activation, antimicrobial defense, epithelial structure, and junctional integrity—not discovery of previously unknown UC genes.

Reviewer #1, comment 2

Comment: Methods: derivation of the 35 barrier-related genes

Response: Section 2.3 now describes the literature and functional categories, search terms, retention logic, symbol harmonization, duplicate handling, detectability check, and the final prespecified set. The complete panel and categories are provided in S3 Table.

Reviewer #1, comment 3

Comment: Methods: normalization, harmonization, and batch effects

Response: Section 2.1 now reports series-matrix processing, probe-to-gene mapping and averaging, transformations/normalization checks, and independent cohort processing. The cohorts were never merged; therefore cross-cohort ComBat correction was not applied. External validation instead used training-derived scaling and a locked model.

Reviewer #1, comment 4

Comment: Results: selection rules, DEFA5, and class imbalance

Response: We explicitly report that DEFA5 passed differential-expression screening but was not retained by the intersection of the LASSO and random-forest priority sets. Inverse-frequency class weights were used during model fitting, and stratification was used for all folds.

Reviewer #1, comment 5

Comment: Discussion: lower GSE38713 performance and generalizability

Response: The Discussion now explains that the lower AUC in GSE38713 may reflect platform, sample-composition, disease-activity, biopsy-location, and cohort-size differences. We emphasize the need for prospective, assay-standardized validation against Crohn’s disease, infectious colitis, and other inflammatory controls.

Reviewer #2 — Major concerns

Reviewer #2, major comment 1

Comment: The term “diagnostic signature” may overstate clinical scope.

Response: We agree. “Diagnostic” was removed from the title and the model is consistently described as a locked public-transcriptomic score/candidate molecular signature. The Abstract, Introduction, Discussion, and Conclusions now emphasize that it has not established UC specificity or clinical readiness.

Reviewer #2, major comment 2

Comment: Clarify the additional biological insight beyond existing signatures.

Response: We no longer claim novel gene discovery. The revised text identifies the contribution as a biologically prespecified framework linking inflammation-associated genes (LCN2/S100A8), antimicrobial defense (DEFB1), mucin/junctional biology (MUC1/TJP3), and epithelial differentiation (VIL1), supported by enrichment and immune-marker correlations.

Reviewer #2, major comment 3

Comment: Verify citations and distinguish the present work from genuinely IBD/UC-focused studies.

Response: We verified all cited references. The GEO database paper was removed from the sentence describing prior UC machine-learning studies, the atrial-fibrillation and atopic-dermatitis articles were deleted, and UC-specific primary studies were cited instead. We also revised the Introduction to distinguish our prespecified 35-gene barrier panel, leakage-aware internal validation, and locked fixed-cutoff external evaluation from prior data-driven or broad-panel approaches. We now explicitly state that the contribution is the barrier-focused analytical organization and validation framework rather than discovery of previously unknown UC genes.

Reviewer #2, major comment 4

Comment: The model may detect nonspecific inflamed, damaged tissue rather than UC.

Response: We agree and elevated this from a secondary limitation to a central caveat throughout the manuscript. The score is interpreted as a pattern associated with UC mucosal inflammation and barrier injury; future evaluation against Crohn’s disease, infectious colitis, and non-IBD inflammatory controls is explicitly required.

Reviewer #2, major comment 5

Comment: Potential information leakage; repeat modeling with nested cross-validation if necessary.

Response: Audit of the original code confirmed that feature selection occurred on the full GSE75214 cohort before cross-validation of the final model. We therefore added 50 repetitions of stratified five-fold outer cross-validation. In the primary locked-score analysis, preprocessing and ridge tuning were confined to each outer-training fold (mean out-of-fold AUC 0.9660, SD 0.0031). In a stricter sensitivity analysis, differential-expression filtering, LASSO, random-forest ranking, and ridge fitting were all repeated within each outer-training fold (mean AUC 0.9600, SD 0.0094). These results and complete code/predictions are supplied.

Reviewer #2 — Minor comments

Reviewer #2, minor comment 1

Comment: Italicize gene symbols throughout.

Response: Gene symbols were standardized and italicized in narrative text and figure labels where typographically appropriate.

Reviewer #2, minor comment 2

Comment: Correct the GSE38713 AUC discrepancy.

Response: All analyses and displays now report AUC 0.8974 (95% CI 0.8040–0.9909) from the locked model.

Reviewer #2, minor comment 3

Comment: Provide higher-resolution Figures 1 and 10.

Response: Both figures were regenerated/provided at publication-quality resolution.

Reviewer #2, minor comment 4

Comment: Test incremental value over VIL1 alone.

Response: A paired DeLong comparison was added. The six-gene AUC (0.9747) was not significantly higher than VIL1 alone (0.9616; P=0.4026). We therefore frame the six-gene score as broader biological representation rather than claiming statistically superior discrimination.

Reviewer #2, minor comment 5

Comment: Verify Figure 7/8 placement and duplication.

Response: The figure set was audited. Figure 7 contains decision-curve analysis; Figure 8 contains immune marker-score heatmap and boxplots. Duplicate/misplaced files were removed from the submission set.

Reviewer #2, minor comment 6

Comment: Remove dashes/underscores in immune-cell names.

Response: Labels were standardized, including “CD4+ T cells” and “CD8+ T cells”; Figures 8A, 8B, and 9 were regenerated.

We again thank the editor and reviewers. We believe the revised manuscript is more transparent, reproducible, and appropriately calibrated to the evidence.

Sincerely,

The Authors

Attachment Submitted filename: Response_to_Reviewers.docx

# Peer review: Decision Letter 1

**DOI:** 10.1371/journal.pone.0361599.r003

**Authors:** Yash Gupta

13 Oct 2026

A Mucosal Barrier Injury Related Six Gene Transcriptomic Signature Associated with Ulcerative Colitis Using Machine Learning and Public Transcriptomic Data

PONE-D-26-35608R1

Dear Dr. he,

We’re pleased to inform you that your manuscript has been judged scientifically suitable for publication and will be formally accepted for publication once it meets all outstanding technical requirements.

Within one week, you’ll receive an e-mail detailing the required amendments. When these have been addressed, you’ll receive a formal acceptance letter and your manuscript will be scheduled for publication.

An invoice will be generated when your article is formally accepted. Please note, if your institution has a publishing partnership with PLOS and your article meets the relevant criteria, all or part of your publication costs will be covered. Please make sure your user information is up-to-date by logging into Editorial Manager at Editorial Manager® and clicking the ‘Update My Information' link at the top of the page. For questions related to billing, please contact billing support.

If your institution or institutions have a press office, please notify them about your upcoming paper to help maximize its impact. If they’ll be preparing press materials, please inform our press team as soon as possible -- no later than 48 hours after receiving the formal acceptance. Your manuscript will remain under strict press embargo until 2 pm Eastern Time on the date of publication. For more information, please contact onepress@plos.org.

Kind regards,

Yash Gupta, Ph.D.

Academic Editor

PLOS One

Additional Editor Comments (optional):

Reviewers' comments:

Reviewer's Responses to Questions

**Comments to the Author**

Reviewer #1: All comments have been addressed

Reviewer #2: All comments have been addressed

**********

2. Is the manuscript technically sound, and do the data support the conclusions?

Reviewer #1: Yes

Reviewer #2: Yes

**********

3. Has the statistical analysis been performed appropriately and rigorously?

Reviewer #1: Yes

Reviewer #2: Yes

**********

4. Have the authors made all data underlying the findings in their manuscript fully available?

The PLOS Data policy

Reviewer #1: (No Response)

Reviewer #2: Yes

**********

5. Is the manuscript presented in an intelligible fashion and written in standard English?

Reviewer #1: Yes

Reviewer #2: Yes

**********

Reviewer #1: All my comments have been meticulously addressed by the authors. This report may be published pending 2nd reviewers comments are addressed.

Reviewer #2: The authors have adequately addressed the major concerns raised in my previous review. In particular, the additional fold-local cross-validation analyses address the potential data-leakage concern, while the revised manuscript appropriately moderates claims regarding diagnostic utility and UC specificity. The remaining limitations are clearly acknowledged and do not substantially detract from the study's conclusions.

Overall, the manuscript has been substantially improved, and I have no further major concerns. I therefore recommend acceptance.

**********

what does this mean?). If published, this will include your full peer review and any attached files.

If you choose “no”, your identity will remain anonymous but your review may still be made public.

**Do you want your identity to be public for this peer review?**  For information about this choice, including consent withdrawal, please see our Privacy Policy

Reviewer #1: **Yes:**  Sandeep Meharwal

Reviewer #2: No

**********

# Editor report: Acceptance letter

**DOI:** 10.1371/journal.pone.0361599.r004

**Authors:** Yash Gupta

PONE-D-26-35608R1

PLOS One

Dear Dr. he,

I'm pleased to inform you that your manuscript has been deemed suitable for publication in PLOS One. Congratulations! Your manuscript is now being handed over to our production team.

At this stage, our production department will prepare your paper for publication. This includes ensuring the following:

* All references, tables, and figures are properly cited

* All relevant supporting information is included in the manuscript submission,

* There are no issues that prevent the paper from being properly typeset

You will receive further instructions from the production team, including instructions on how to review your proof when it is ready. Please keep in mind that we are working through a large volume of accepted articles, so please give us a few days to review your paper and let you know the next and final steps.

Lastly, if your institution or institutions have a press office, please let them know about your upcoming paper now to help maximize its impact. If they'll be preparing press materials, please inform our press team within the next 48 hours. Your manuscript will remain under strict press embargo until 2 pm Eastern Time on the date of publication. For more information, please contact onepress@plos.org.

You will receive an invoice from PLOS for your publication fee after your manuscript has reached the completed accept phase. If you receive an email requesting payment before acceptance or for any other service, this may be a phishing scheme. Learn how to identify phishing emails and protect your accounts at https://explore.plos.org/phishing.

If we can help with anything else, please email us at customercare@plos.org.

Thank you for submitting your work to PLOS One and supporting open access.

Kind regards,

PLOS One Editorial Office Staff

on behalf of

Dr. Yash Gupta

Academic Editor

PLOS One
